# Cryo-ET of Env on intact HIV virions reveals structural variation and positioning on the Gag lattice

**DOI:** 10.1016/j.cell.2022.01.013

**Published:** 2022-02-17

**Authors:** Vidya Mangala Prasad, Daniel P. Leaman, Klaus N. Lovendahl, Jacob T. Croft, Mark A. Benhaim, Edgar A. Hodge, Michael B. Zwick, Kelly K. Lee

**Affiliations:** 1 Department of Medicinal Chemistry, University of Washington, Seattle, WA 98195, USA; 2 Biological Physics, Structure and Design Graduate Program, University of Washington, Seattle, WA 98195, USA; 3 Department of Microbiology, University of Washington, Seattle, WA 98195, USA; 4 Department of Immunology and Microbiology, The Scripps Research Institute, La Jolla, CA 92037, USA

**Keywords:** HIV Env glycoprotein, HIV assembly, Gag-Env interaction, cryo-electron tomography, sub-tomogram averaging, hydrogen/deuterium-exchange mass spectrometry, broadly neutralizing antibody, cryo-electron microscopy, virus structure, vaccine design

## Abstract

HIV-1 Env mediates viral entry into host cells and is the sole target for neutralizing antibodies. However, Env structure and organization in its native virion context has eluded detailed characterization. Here, we used cryo-electron tomography to analyze Env in mature and immature HIV-1 particles. Immature particles showed distinct Env positioning relative to the underlying Gag lattice, providing insights into long-standing questions about Env incorporation. A 9.1-Å sub-tomogram-averaged reconstruction of virion-bound Env in conjunction with structural mass spectrometry revealed unexpected features, including a variable central core of the gp41 subunit, heterogeneous glycosylation between protomers, and a flexible stalk that allows Env tilting and variable exposure of neutralizing epitopes. Together, our results provide an integrative understanding of HIV assembly and structural variation in Env antigen presentation.

## Highlights

• Structural analysis of immature HIV shows Env position on Gag hexameric rim
• HIV Env has a flexible stalk that allows tilting and variation in stalk exposure
• Env’s fusion peptide is dynamic and exposed to solvent in membrane-bound Env
• Glycans in unliganded Env shield antigenic sites and vary between protomers

## Graphical abstract

**Figure:**
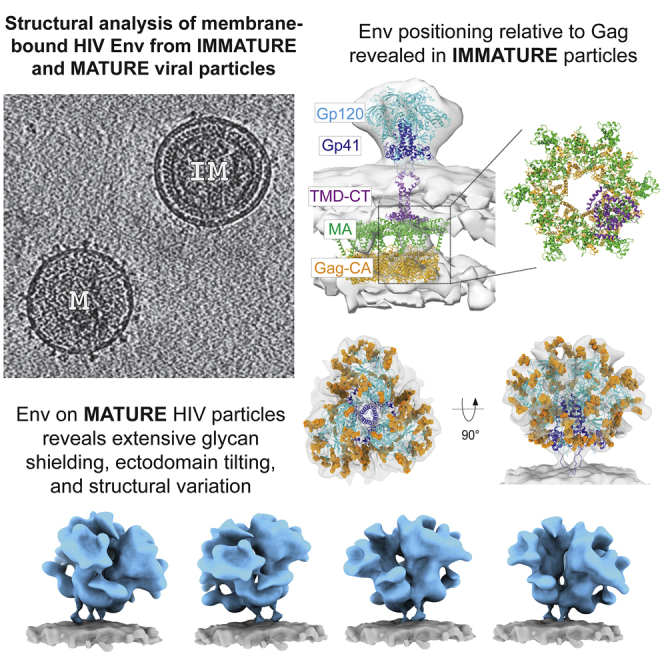

## Teaser

Looking at native HIV virions using cryo-ET shows how Env interacts with Gag and reveals variability in Env conformations that is relevant for understanding interactions with antibodies.

## Introduction

Human immunodeficiency virus-1 (HIV-1) continues to infect nearly two million people worldwide each year, with no vaccine available against the virus (Pandey and Galvani, 2019). The envelope glycoprotein (Env) on the surface of HIV-1 is an essential viral entry machine that mediates binding to host cell receptors and subsequent membrane fusion. As the sole target for neutralizing antibodies, Env is also of singular importance for vaccine design efforts (van Gils and Sanders, 2013). Env is translated as a precursor gp160 protein, which trimerizes and is cleaved into gp120 and gp41 subunits that are primarily responsible for receptor binding and fusion, respectively. The gp120 and gp41 subunits remain as non-covalently associated heterodimers with gp41 embedded in the viral membrane via its transmembrane domain (TMD) and the C-terminal cytoplasmic domain (CTD) in the viral lumen. Upon incorporation into budding virions, Env CTD interacts with the matrix domain (MA) of immature Gag polyprotein, which assembles as a membrane-associated lattice on the inner side of the viral bilayer (Checkley et al., 2011; Tedbury and Freed, 2014).

Recent insights into Env structure have primarily come from studies of engineered, truncated forms of the trimer ectodomain (such as SOSIPs) (Stewart-Jones et al., 2016; Ward and Wilson, 2015), while details of Env structure in its viral membrane context, including its organization relative to other viral components, have remained elusive. In order to understand how Env is assembled on virions and how it differs structurally from soluble trimers, we carried out cryo-electron tomography (cryo-ET) with sub-tomogram averaging and structural mass spectrometry analysis of Env displayed on immature and mature Gag-bearing HIV-1 virus-like particles (VLPs). These studies advanced our knowledge of membrane-bound Env via insights gained from cryo-ET of intact viral particles.

## Results and discussion

**Figure 1:**
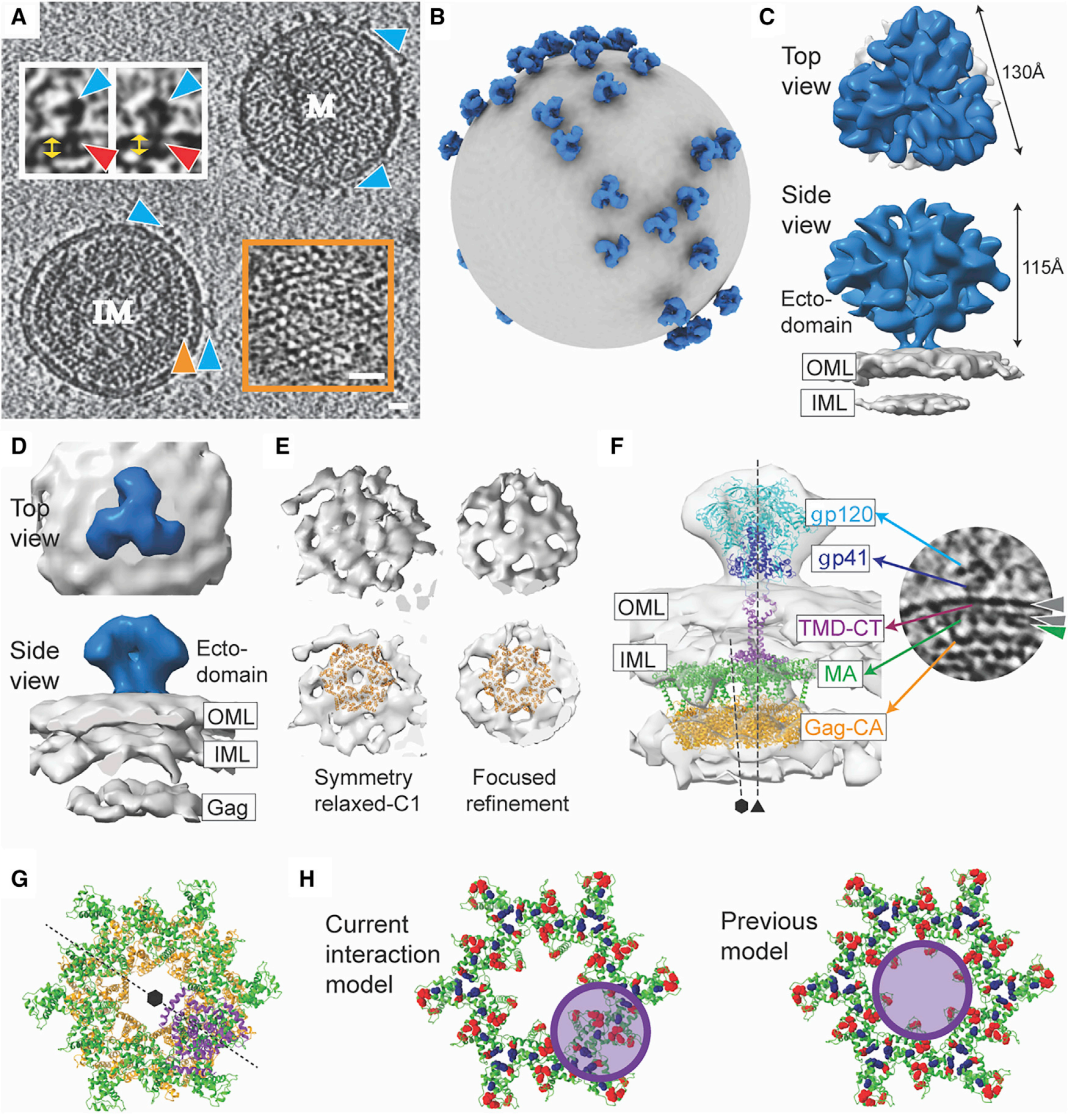
Structural analysis of hVLP-Env (A) Tomogram slice showing a mature (M) and immature (IM) hVLP. Blue arrows indicate Env. Orange arrow indicates Gag layer in IM with zoomed inset of Gag top view on bottom right. Scale bars indicate 200 Å. Top left inset shows close-up of Env with red arrows denoting TMD and yellow arrows denoting membrane bilayer. For antigenic profile of hVLPs, see Figures S1 and S2 and Table S1. (B) Representative model of mature hVLP with averaged Env structure (in blue) placed onto original particle coordinates. Membrane surface is shown in gray. (C) Sub-tomogram-averaged Env structure at 9.1Å resolution (for resolution-related statistics, see Figure S3). OML and IML indicate outer and inner membrane leaflets, respectively. (D) Sub-tomogram-averaged Env structure from immature VLPs. OML and IML indicate outer and inner membrane leaflets, respectively. (E) Top views of immature Gag layer from relaxed C1 symmetry map (top left) and after focused refinement (top right) (see also Figure S3). Bottom panels show the same with fitted atomic structure of hexameric Gag-CA (PDB: 4USN). (F) Left: composite model of Env-Gag structures in immature particles. Atomic coordinates of Env ectodomain with gp120 (cyan) and gp41 (dark blue), TMD and CTD in purple (PDB: 6UJV), Gag-MA hexamer (PDB: 1HIW) in green, and Gag-CA hexamer in orange are shown. Right: tomogram slice of Env from an immature particle with protein components labeled. Grey and green arrows indicate membrane bilayer and Gag-MA, respectively. (G) Top view of CTD (purple) and Gag-MA (green) interface. CTD lies on the rim of underlying Gag-CA hexamer (orange). (H) Model of Env-CTD interaction with Gag-MA. Ribbon diagram of Gag-MA hexamer (green) (PDB ID: 1HIW) (Hill et al., 1996) is shown. Residues that reduce Env incorporation when mutated (13L, 17E, 31L, 35V, 99E, and 75LG) are highlighted as red spheres (Alfadhli et al., 2019). Residues that reportedly suppress Env incorporation defects (62QR, 35VI, and 44FL) are shown as blue spheres (Alfadhli et al., 2019). Position and area covered by Env CTD structure (based on PDB ID: 6UJV [Piai et al., 2020]) in our current model (left) and previously predicted models (right) are shown as purple circle. In previous models (right panel), Env CTD position was predicted to be at the center of the Gag-MA hexamer. In this configuration, Env CTD directly interacts with only a few residues that line the inner circumference of Gag-MA hexamer. Thus, the effect of the majority of involved residues on Env incorporation would be allosteric. In the current model (left panel) based on our cryo-ET data, the Env CTD makes direct contacts with all Gag-MA residues determined to affect Env incorporation.

**Figure S1:**
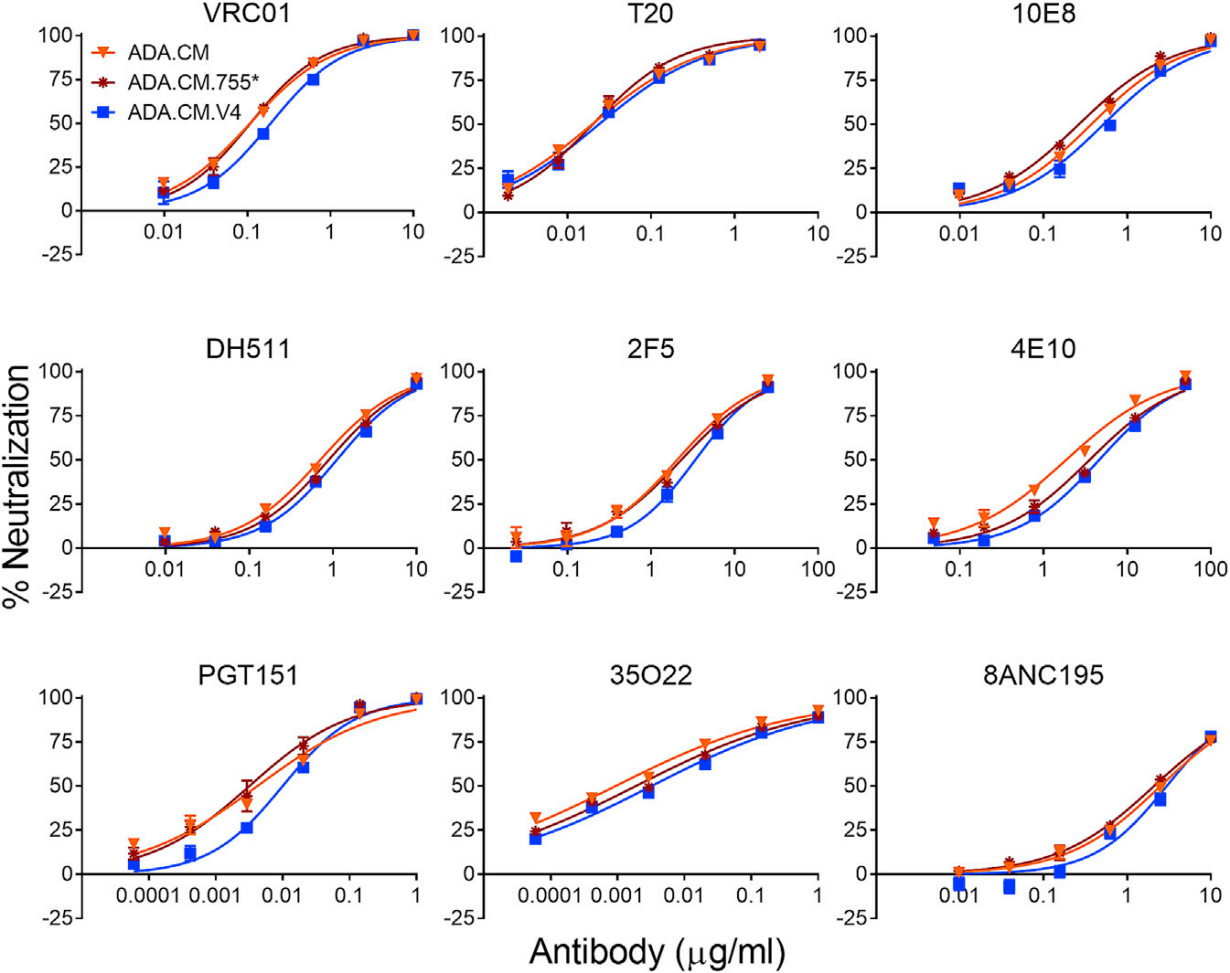
Percentage neutralization of HIV particles using a multi-antibody panel and the gp41 fusion inhibitor peptide, T20, related to Figure 1 ADA.CM and ADA.CM.755^∗^ denote pseudotyped HIV particles produced by transient transfection of HEK293T cells using a complementary two-plasmid method, with one plasmid DNA bearing the HIV backbone genes except Env, and the other plasmid bearing either Env displaying full length ADA.CM Env, or ADA.CM with a CTD truncation similar to that in hVLPs (stop codon at amino acid 756), respectively. ADA.CM.V4 refer to hVLPs produced by transient transfection of the ADA.CM.V4 high-Env HEK293T stable cell line with plasmid DNA bearing the HIV backbone genes except Env.

**Figure S2:**
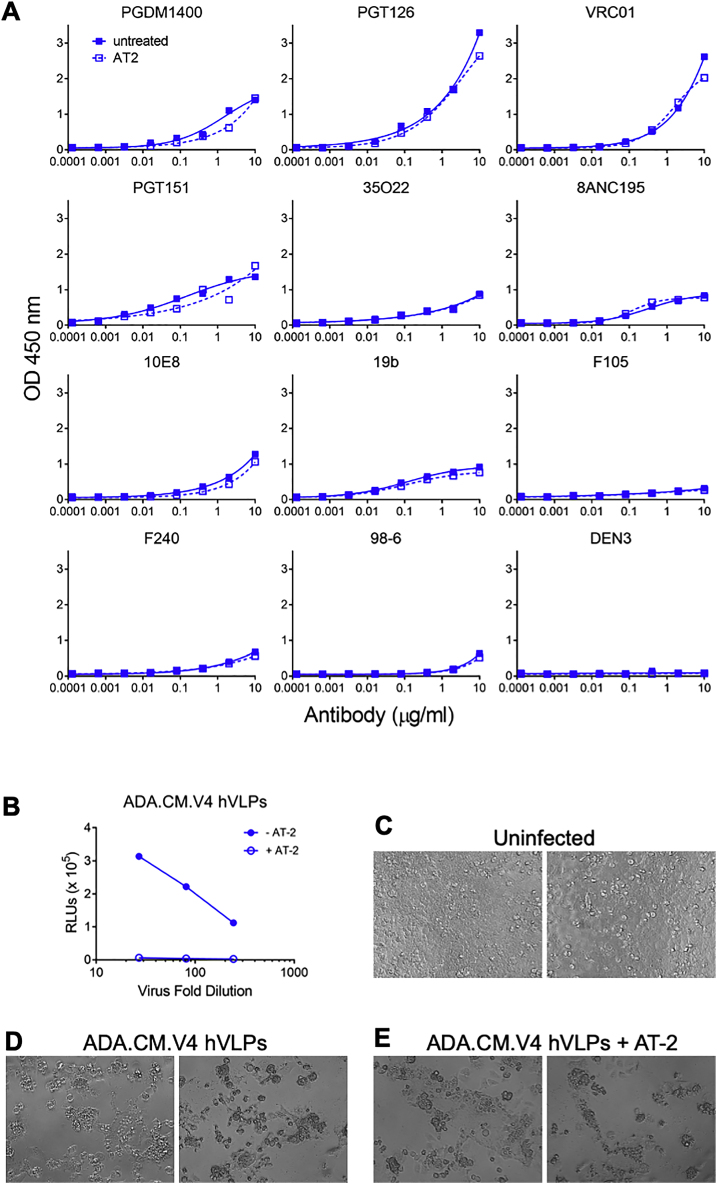
Effect of AT-2 treatment on hVLPs, related to Figure 1 (A) Binding of antibodies to hVLPs, as probed using an ELISA. hVLPs were treated in presence (open squares) or absence (closed squares) of AT-2 and directly immobilized on microwell plates. Binding curves show no significant difference in antibody binding on treatment with AT-2, indicating that the antigenic surface of hVLP-Env has not been altered due to AT-2 inactivation. (B) ADA.CM.V4 hVLPs, treated in presence (open circles) and absence (close circles) of AT-2 and normalized for p24 content, were overlaid on TZM-bl reporter cells and luciferase expression was monitored 72 h later. AT-2-treated hVLPs do not cause productive infection. (C–E) Cells that had been incubated with hVLPs as in (B) were imaged using a light microscope and representative images are shown. Uninfected cells are monodispersed, cells incubated with hVLPs (treated either in presence or absence of AT-2) induce syncytia formation and cytotoxicity. Images are from wells in which hVLP containing supernatants were effectively diluted 3-fold prior to addition to cells.

**Figure S3:**
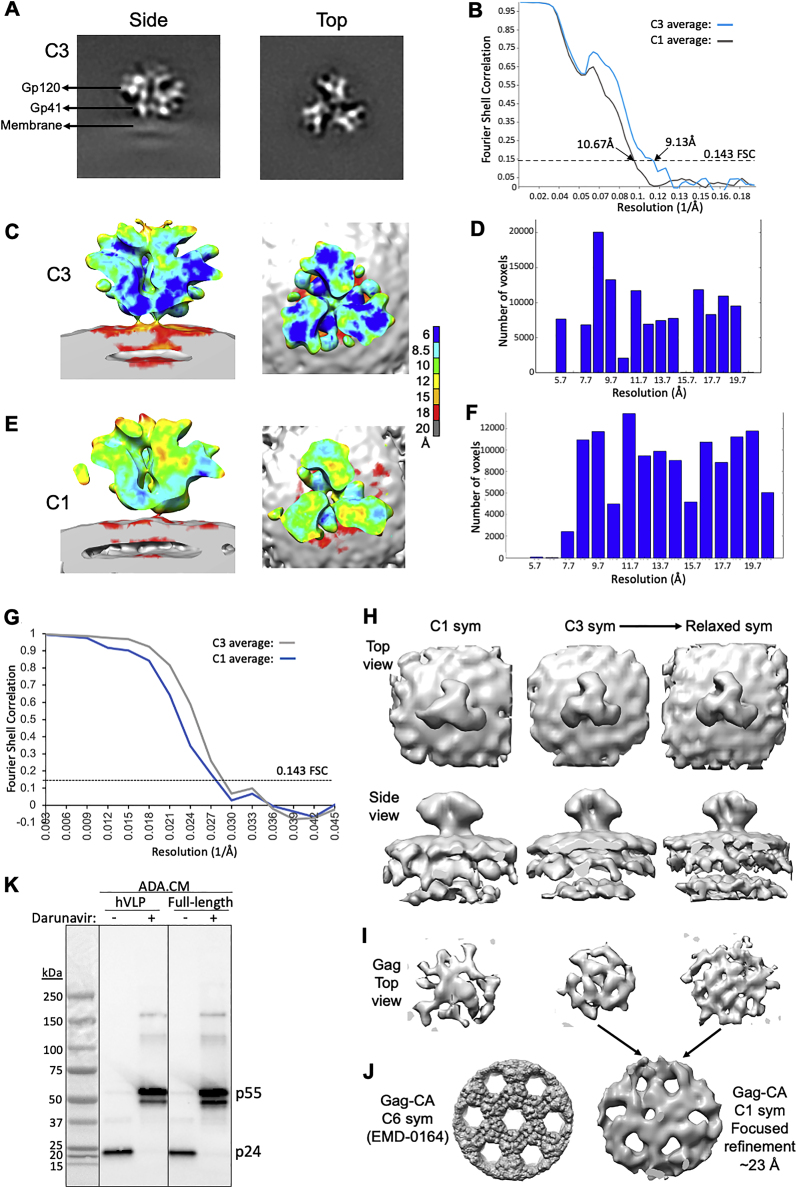
Structural analyses of Env sub-tomogram averaged maps from mature and immature hVLPs, related to Figures 1, 2, 3, and 5 (A) Perpendicular slices through the C3-symmetrized hVLP-Env map. (B) Gold-standard Fourier shell correlation (FSC) curves for C3-symmetrized map (blue line) and C1-symmetry map (gray line). Dotted line indicated the 0.143 FSC cutoff. (C–F) Local resolution estimation using Resmap software (Kucukelbir et al., 2014) for C3-symmetrized map (C and D) and asymmetric map (E and F). (G) Fourier shell correlation (FSC) curves for C3 (gray line) and C1 (blue line) symmetrized maps of immature hVLP-Env. (H) Top and side surface views of immature hVLP-Env structure with C1 symmetry, C3 symmetry and the C3 map relaxed to C1 symmetry. (I) Top view of Gag layer from corresponding averages in panel B. (J) Left: Previously published high resolution density map of Gag-CA lattice, low pass filtered to ∼10 Å (Mattei et al., 2018). Right: Gag-CA structure derived after focused refinement of Gag layer from the relaxed C1 symmetry immature Env map. (K) HIV protease inhibitor darunavir blocks the processing of immature pr55 Gag precursor into the mature p24 capsid protein. ADA.CM Env with 102 residues truncation in CTD from hVLPs and full-length Env-CTD from pseudovirus, produced in the presence (+) or absence (-) of darunavir, were separated by SDS-PAGE and probed by western blot staining with an anti-p24 antibody.

Virion particles bearing ADA.CM Env, a variant of subtype B isolate ADA that was selected for stability (Leaman and Zwick, 2013), were produced in human HEK293T cells as previously described (Figures 1A and 1B) (Stano et al., 2017). These membrane-enveloped VLPs, referred to as high Env VLPs or hVLPs, display elevated levels of Env trimers that are fully functional for mediating receptor binding and entry. Env on hVLPs retain the antigenic profile of full-length Env (Figure S1; Table S1), despite a C-terminal truncation of 102 amino acids in the CTD (Stano et al., 2017). hVLPs assemble around a functional Gag layer that undergoes proteolytic maturation and have the composition of authentic HIV-1 virions but are replication incompetent because the packaged genome lacks the Env gene (Leaman and Zwick, 2013; Stano et al., 2017). Purified hVLPs were additionally treated with aldrithiol-2 (AT-2), a mild oxidizing agent that interrupts nucleocapsid interaction with RNA to ensure non-infectivity (Rossio et al., 1998). Antibody binding experiments showed that the antigenicity of hVLP-Env was unaffected by AT-2 treatment (Figure S2). Despite the lack of infectivity of AT-2-treated hVLPs, they induced syncytia formation and cytotoxicity in cells, similar to non-AT-2-treated hVLPs, in accordance with a previous report (Rossio et al., 1998), showing that AT-2-treated HIV-1 virions bear functional Envs that are capable of mediating cell entry (Figure S2). These results indicate that the Env conformation on the hVLPs was not significantly altered by AT-2, as the antigenicity and viral entry properties of Env are retained. The high density of surface Env on hVLPs allowed us to gather sufficient cryo-ET data to obtain a 9.1 Å resolution structure of Env in a membrane context on these VLPs, which we discuss further below (Figures 1C and S3).

### Env structure from immature particles reveal interactions with Gag

Though the hVLPs in our sample population were predominantly mature, approximately 3% of the particles appeared immature, exhibiting a clear, assembled Gag lattice beneath the viral membrane (Figure 1A). Env subtomogram volumes from only these immature particles were averaged together to give density maps at ∼31 Å (C3 symmetry) and ∼34 Å (C1 symmetry) (Figures 1D and S3G). The Env structure from immature particles closely resembles the subnanometer Env structure generated from the total hVLP population (Figure 1C) and other reported Env ectodomain structures (Kwon et al., 2015; Ward and Wilson, 2015). In the averaged immature Env map, a third density layer is seen underneath the membrane bilayer corresponding to the Gag lattice position (Figures 1A, 1D, S3H, and S3I). Focused refinement of this internal third layer revealed a distinct structure that clearly resembles a lattice formed by the capsid domain (CA) of immature Gag polyprotein (Figures 1E and S3J) (Schur et al., 2015). Structures of trimeric Env ectodomain (Pan et al., 2020) and hexameric Gag-CA (Schur et al., 2015) were fitted as rigid bodies into corresponding densities in the averaged map (Figure 1F). No clear density for the Env TMD-CTD was observed in the averaged structure, even though the TMD is seen in individual raw tomograms (Figure 1F), suggesting that the TMD is not strictly aligned with the ectodomain or Gag-CA. The structure of the TMD-CTD (PDB ID: 6UJV) was thus placed into the map based on its position relative to the Env ectodomain (Figure 1F) (Piai et al., 2020).

**Figure 2:**
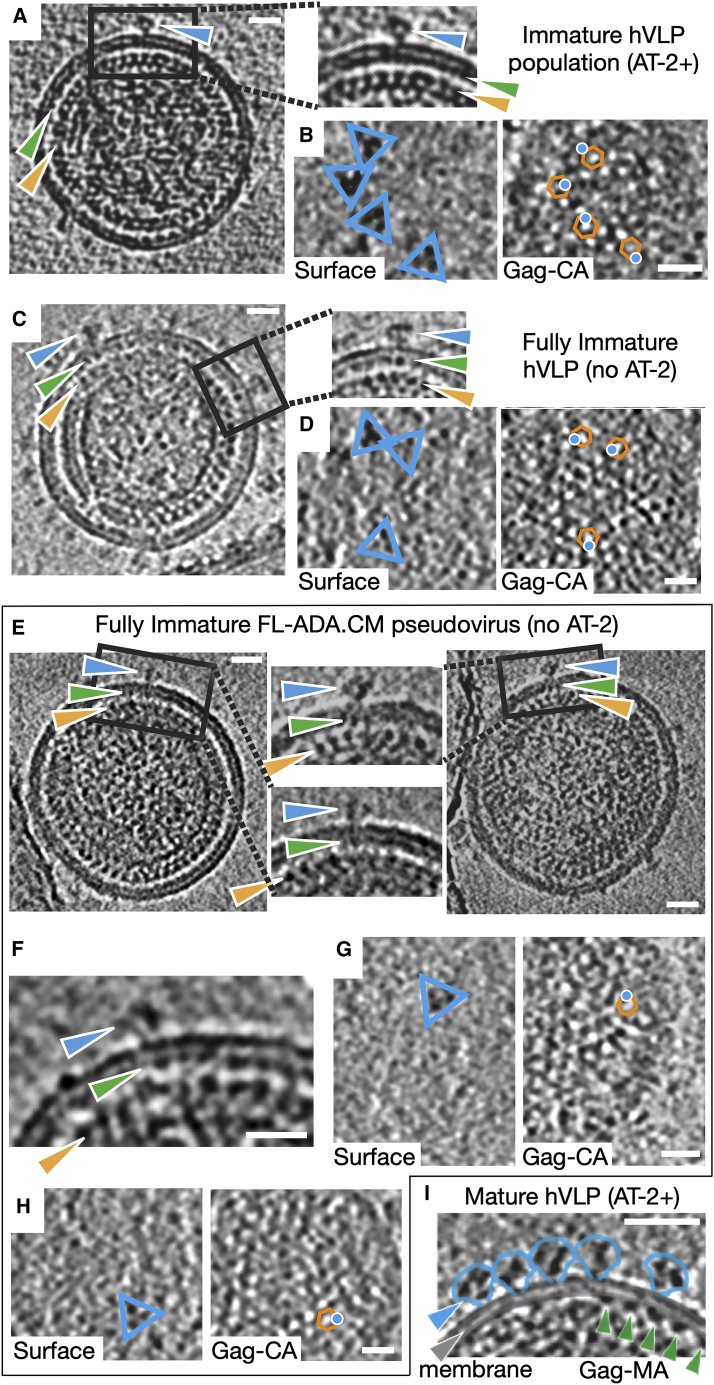
HIV Env positioning over the immature Gag lattice (A and B) Representative immature sub-population of AT-2-treated ADA.CM.v4 hVLP particles. (A) Tomogram slices through central section showing position of Env (blue arrow) over MA (green arrow) and CA (orange arrow). (B) Top view of VLP surface plane and through Gag-CA layer. Env particles are indicated by blue triangles (left panel). Right panel shows position of Env central axis (blue dot) with respect to the Gag-CA hexamer (orange hexagon) when viewing along an axis perpendicular to the plane of image. (C and D) Representative fully immature, non-AT-2-treated ADA.CM.v4 hVLP particles. Tomogram slices through central section (C), at VLP surface plane (D [left]), and through Gag-CA layer (D [right]). (E–H) Representative fully immature, non-AT-2-treated full-length ADA.CM Env bearing VLP particles. Tomogram slices through central section (E and F) and at VLP surface plane (G and H [left]) and through Gag-CA layer (G and H [right]). (D), (G), and (H) are all annotated in a similar way as (B). Notably, in all of them, Env is positioned on the rim of the Gag hexamer. Black represents high density in all panels. Tomogram slice thickness is 10.32 Å in (A) and (B) and 8.32 Å in (C)–(H). (I) Mature ADA.CM.v4 hVLP showing loss of Env (blue)/Gag-MA (green) colocalization in cross-sectional central slice. Slice thickness is 10.32 Å. Scale bars are 200 Å in length.

The N-terminal MA domain of immature Gag polyprotein assembles along the inner membrane leaflet as a hexameric lattice of trimers (Frank et al., 2015; Qu et al., 2021; Tedbury et al., 2016) and is connected by a flexible linker to the Gag-CA lattice underneath (Tedbury and Freed, 2014). Regularly spaced puncta corresponding to the Gag-MA domain can be discerned directly underneath the inner leaflet and Env ectodomains in immature hVLP tomograms (Figures 1F and 2A). However, distinguishable density for the Gag-MA layer was not seen in the averaged immature map, implying that the Gag-MA lattice (Frank et al., 2015; Tedbury and Freed, 2014; Tedbury et al., 2016) may have subtle variations in arrangement that deviate from Gag-CA or Env symmetry axes. Hence, based on the expected position of Gag-MA relative to the Gag-CA lattice (Frank et al., 2015; Tedbury et al., 2016), the MA lattice structure was placed into the map (Figure 1F). The resulting juxtaposition of Env CTD and Gag-MA occurs right at the bottom of the inner membrane as would be expected from the raw tomograms (Figures 1F and 2). From this composite model, the relative position of Env CTD, when viewed normal to the membrane, is on the rim of the hexameric Gag-CA lattice along its 2-fold axis (Figure 1G). This observation can also be corroborated in the raw tomograms (Figures 2A and 2B). Thus, the trimeric Env CTD does not lie in the central hole formed by the immature Gag lattice as has been hypothesized (Tedbury and Freed, 2014; Tedbury et al., 2016) but instead is positioned on its hexameric rim (Figures 1G and 1H). In this configuration, the modeled Env CTD makes direct contact with critical residues on MA that were shown previously to be necessary for Env incorporation in virions (Figure 1H) (Tedbury and Freed, 2014; Tedbury et al., 2016).

To confirm that the observed Env positioning relative to Gag-CA in immature hVLPs is not affected by AT-2 treatment, lack of a complete C-terminal tail, or incomplete processing of Gag precursor, we collected cryo-ET data of fully immature hVLPs displaying ADA.CM trimers and immature VLPs displaying full-length ADA.CM trimers, without AT-2 treatment in both cases. These fully immature VLPs were produced in the presence of protease inhibitor darunavir such that the resulting particles have greater than 95% unprocessed Gag (Figure S3K). Analysis of cryo-electron tomograms from these immature VLPs having unprocessed Gag polyprotein further confirmed that the organization of the Gag polyprotein, especially the MA and CA domains, is similar to that seen in the AT-2-treated immature hVLPs (Figure 2). Additionally, Env trimers are indeed positioned along the rim of the Gag-CA lattice and not in the central hole of the Gag-CA hexamers (Figures 2D, 2G, and 2H), identical to that observed in the AT-2-treated hVLP sample. Preservation of this Env-Gag colocalization in the presence of truncated as well as complete cytoplasmic tail suggests that a subset of CTD residues in the baseplate may be sufficient for mediating interactions with Gag, but the remainder of the tail may reduce incorporation into particles through steric hinderance with the Gag lattice (Buttler et al., 2018).

### Rearrangement of MA layer occurs during maturation

In immature particles, the Gag-MA layer is consistently arrayed underneath the inner membrane (Figures 1A, 1F, 2A, 2C, 2E, and 2F), whereas in mature particles where the Gag polyprotein has been proteolytically cleaved, the MA layer appears fragmented (Figure 2I), indicating that MA rearranges during particle maturation, consistent with a recent report (Qu et al., 2021). Notably, Env trimers in the mature particles are no longer colocalized with the MA layer. Given this ultrastructural reorganization following maturation, it seems likely that disruption of membrane-associated Gag lattices may be a prerequisite for Env trimers to gain necessary mobility to mediate membrane fusion (Chojnacki et al., 2012; Roy et al., 2013; Wyma et al., 2004). These structural changes may also provide sufficient membrane pliability for remodeling during viral fusion, similar to matrix layer disruptions seen in other viruses such as influenza virus (Gui et al., 2016).

### Sub-nanometer HIV-1 Env structure shows a conformationally variable gp41 subunit

**Figure 3:**
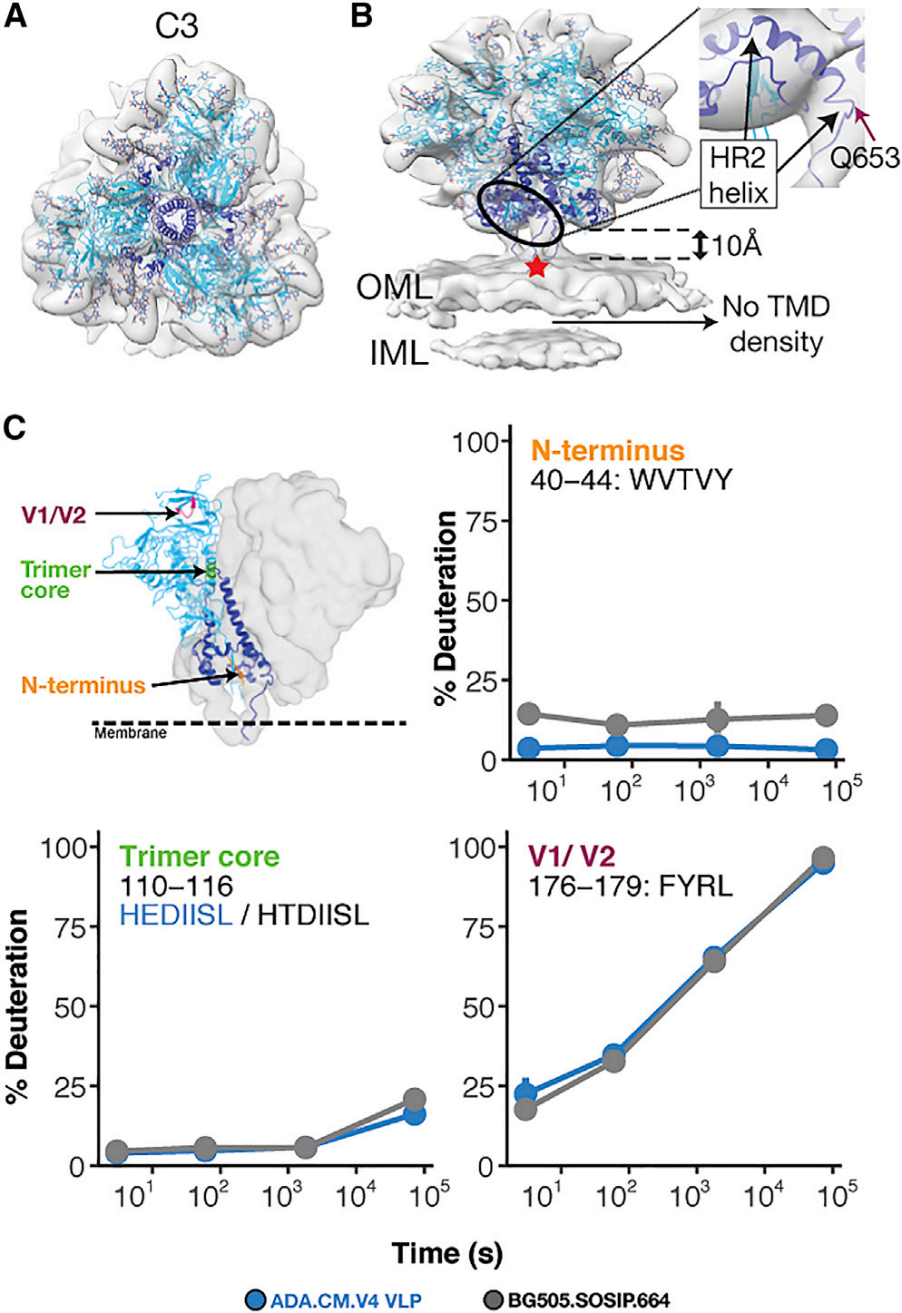
Sub-nanometer structure of hVLP-Env indicates conformational variation in HR2 helix and MPER location (A and B) Top (A) and side-view (B) of sub-tomogram-averaged Env map with fitted ribbon structure adapted from full-length Env structure (PDB ID: 6ULC). gp120 subunit is colored in cyan, gp41 in dark blue, and glycans are colored as red and blue heteroatoms. Outer and inner membrane leaflets are indicated as OML and IML, respectively. Zoomed inset shows the shortened HR2 helix fitted into its respective density. Red star indicates position of Asp-664 in the trimer structure. See also Figure S6. (C) HDX-MS of hVLP-Env shows protection of peptides that report on the closed prefusion conformation of HIV-1 Env (Guttman et al., 2014, 2015; Guttman and Lee, 2013) (see also Figures S4 and S5). Deuterium uptake for peptides reporting on trimer integrity behave similarly between hVLP-Env and BG505.SOSIP, which predominantly samples a closed prefusion conformation.

A total of 32,802 sub-volumes were used to reconstruct a C3-symmetric sub-tomogram-averaged structure of hVLP-Env with local resolution ranging between 5.7–12 Å and a global resolution of 9.1 Å (Figures 1C, 3A, 3B, and S3; Table 1). Classification of the sub-volumes did not yield any other distinct conformational class, indicating that the hVLP-Env were in a predominantly closed, pre-fusion state. The Env ectodomain connects to the membrane via a thin tripod stalk (Figures 1C and 3B). Though the TMD can be observed spanning the membrane layer in raw tomograms (Figure 1A), no evidence of TMD is seen in the sub-tomogram-averaged structure (Figure 3B), similar to the Env structure from immature hVLPs.

**Table 1:** Data collection and processing parameters for cryo-ET and sub-tomogram averaging

Data collection and processing parameters				
**Collection parameters**				

Magnification	58,000×			
Voltage	300 kV			
Pixel size	2.58 Å/pixel			
Electron dose	64–68 e^−^/Å^2^			
Defocus range (μm)	−2.5 to −5.0			
Total number of tilt-series	526			
Final number of tilt-series	423			

**Processing parameters**				

Symmetry form of hVLP-Env	C3 symmetry (sym)	C1 sym	Immature C3 sym	Immature C1 sym
Initial number of sub-volumes	63,592	63,592	1,520	1,520
Final number of sub-volumes	32,802	29,074	1,051	1,059
Map resolution at 0.143 FSC	9.13 Å	10.67 Å	31.35 Å	34.3 Å

Env copy number on individual mature hVLP particles ranged from 8–82 Env, and the trimers appeared to be randomly distributed with no apparent clustering or predisposition to forming close interactions with other trimers (Figure 1B). The hVLPs have a predominantly spherical morphology in our sample with an average diameter of ∼100 nm. Thus, by calculating the surface area of the VLPs as a sphere and the Env trimer as an equilateral triangle with sides of 13 nm length, we can determine that an average hVLP can accommodate up to 429 Env trimers (31,400 nm^2^/73.18 nm^2^) without clashing. Thus, although hVLPs display high levels of Env compared to wild-type HIV, the number of surface Env trimers present on hVLPs was much lower than the theoretical maximum, so Env trimers were not cramped and have sufficient surface area for mobility. Furthermore, the sub-volumes used in the final reconstruction of hVLP-Env were also selected to exclude neighboring Env in close proximity to avoid any influence due to unlikely clashing.

**Figure S4:**
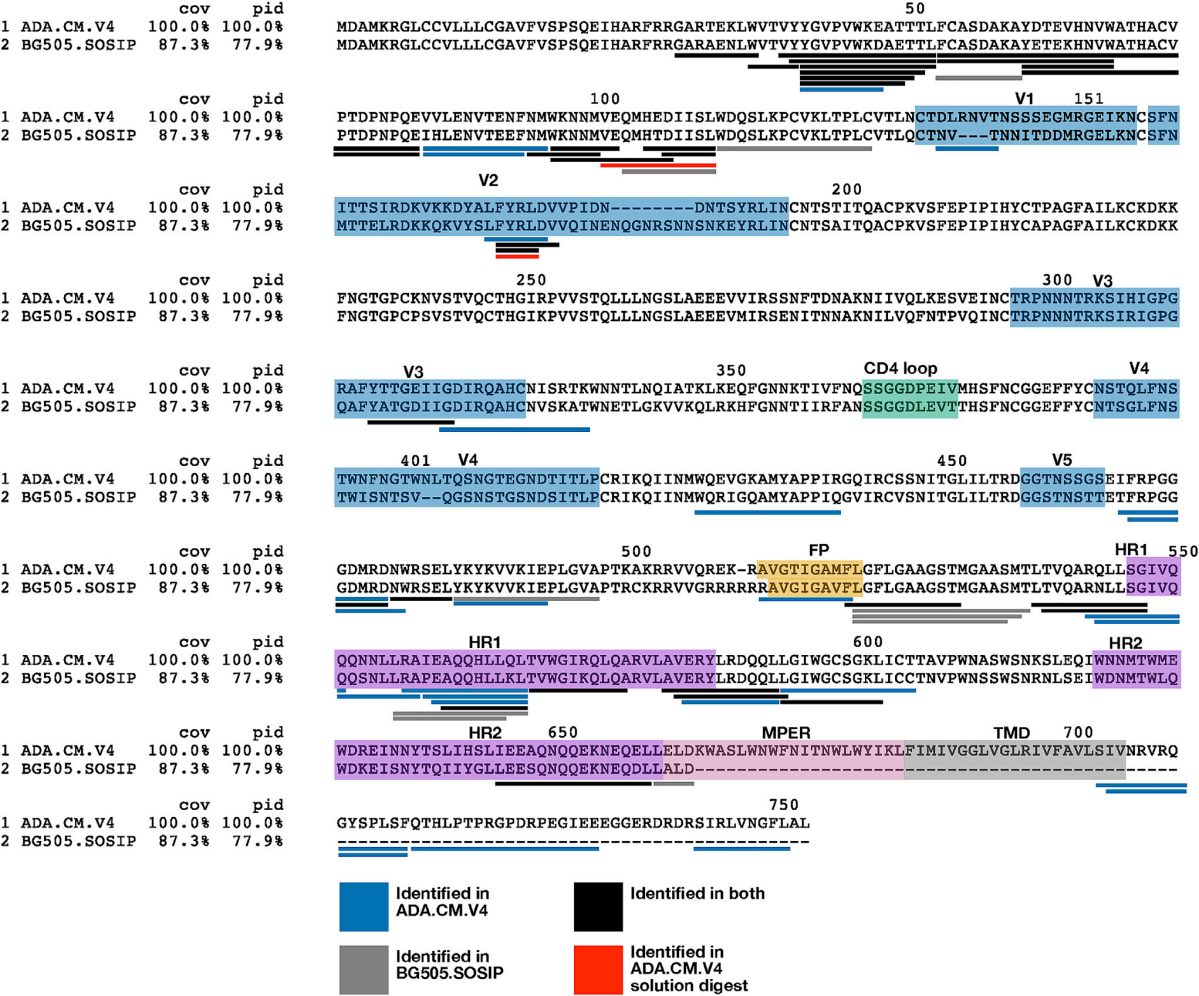
Hydrogen-deuterium exchange mass spectroscopy (HDX-MS) peptide coverage for hVLP-Env analysis, related to Figures 3 and 5 Peptide coverage map of hVLP-Env by quench lysis HDX-MS. Sequence alignment of ADA.CM and BG505.SOSIP used in the HDX-MS experiments. BG505.SOSIP includes residues only up to 664 and does not contain MPER, TMD or CTT domains. Underlined peptide regions are color coded based on their identification in the quench lysis HDX-MS experiment. Peptides underlined in red are those identified by solution digest HDX-MS. Important regions of Env such as variable loops (V1-V5), CD4 receptor binding loop, fusion peptide (FP), heptad repeat helices (HR1 and HR2), MPER and TMD are highlighted and labeled.

**Figure S5:**
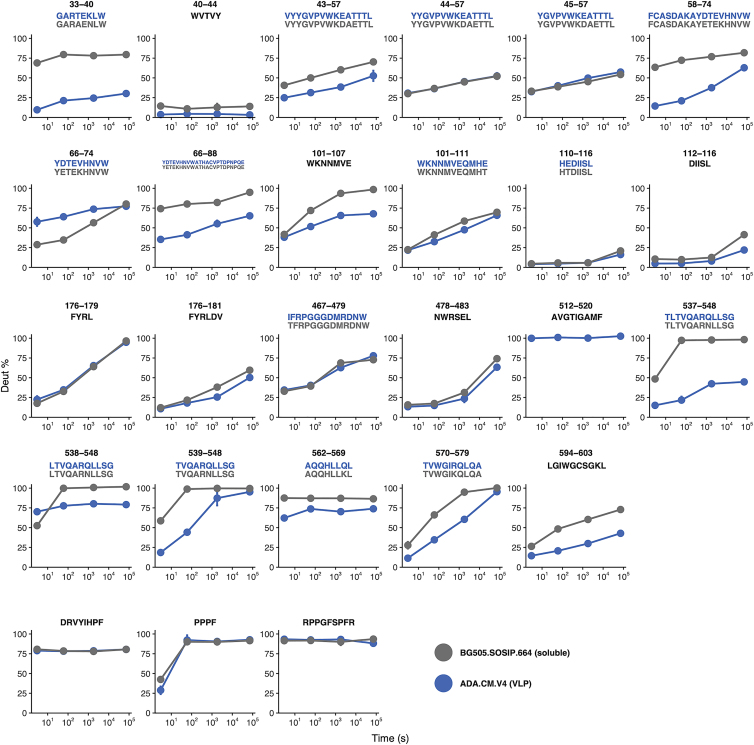
Deuterium uptake plots from HDX-MS experiment, related to Figures 3 and 5 Deuterium uptake plots for all peptides in Env ectodomain commonly identified between hVLP-Env (blue) and BG505.SOSIP (gray). Corresponding sequence of the peptides are given above each uptake plot. Bottom three panels are back-exchange peptide standards (DRVYIHPF and RPPGFSPFR) and exchange rate peptide standard (PPPF). Refer to Figure S4 for sequence alignment between the two Envs.

To gain complementary structural insight into membrane-embedded Env, we conducted hydrogen-deuterium exchange mass spectrometry (HDX-MS) analysis of Env from intact hVLPs. This approach samples the entire population of Env in our specimen. HDX-MS analysis showed that key fiducial peptides that report on trimer integrity (Guttman et al., 2014, 2015; Guttman and Lee, 2013) were well-protected in hVLP-Env (Figure 3C), confirming its closed, pre-fusion conformation. Moreover, the HDX-MS spectra were unimodal, consistent with a homogeneous conformational population of Env. Relative to standard HDX-MS experimental approaches, we were able to improve peptide coverage by performing deuterium exchanges under native conditions but solubilizing the surface protein using detergent under quench conditions. Comparison of hVLP-Env with BG505.SOSIP showed that cognate peptides throughout the trimers had similar exchange profiles (Figures S4 and S5), indicating that they both have comparable global conformations. However, the hVLP-Env trimer on virions exhibited greater exchange protection, indicating it is in general more structurally ordered than the engineered soluble trimer (Figures 3C and S5). Indeed, in terms of gp120 subunit organization, hVLP-Env has a global architecture that closely resembles previously published Env structures (Kwon et al., 2015; Lee et al., 2016; Pan et al., 2020; Ward and Wilson, 2015). This is confirmed by good agreement seen in fitting the atomic structure of detergent-extracted full-length Env (PDB ID: 6ULC) into the sub-tomogram-averaged map (Figures 3A and 3B).

### Differences in gp41’s terminal HR2 helix and stalk influence MPER surface exposure

Significant differences relative to available structures are evident, however, in the gp41 subunit of hVLP-Env. In nearly all known Env structures, the HR2 helix has a long, rod-like conformation with Asp664 forming its distal tip (Kwon et al., 2015; Ward and Wilson, 2015). In contrast, in hVLP-Env, Gln-653 forms the distal tip of the HR2 helix, making the helix length 10 amino acids shorter (Figure 3B). The remaining residues in the C-terminal portion of HR2 helix bend and form a thin stalk connecting the ectodomain to the membrane (Figure 3B). Apart from hVLP-Env, this bend in HR2 helix has only been observed in the full-length, detergent-solubilized Env structure derived from strain 92UG037.8 (Pan et al., 2020). In the presence of membrane, we observe that the thin stalks, corresponding to Env residues 654–664, form a tripod that elevates the ectodomain ∼10 Å above the membrane (Figure 3B). As a result, the bulk of the membrane-proximal external region (MPER) (residues 660–683), which is a desirable vaccine target of some of the most broadly neutralizing antibodies (bnAbs) of HIV-1 isolated to date (Gray et al., 2009; Rantalainen et al., 2020), is primarily embedded within the membrane in our structure.

**Figure S6:**
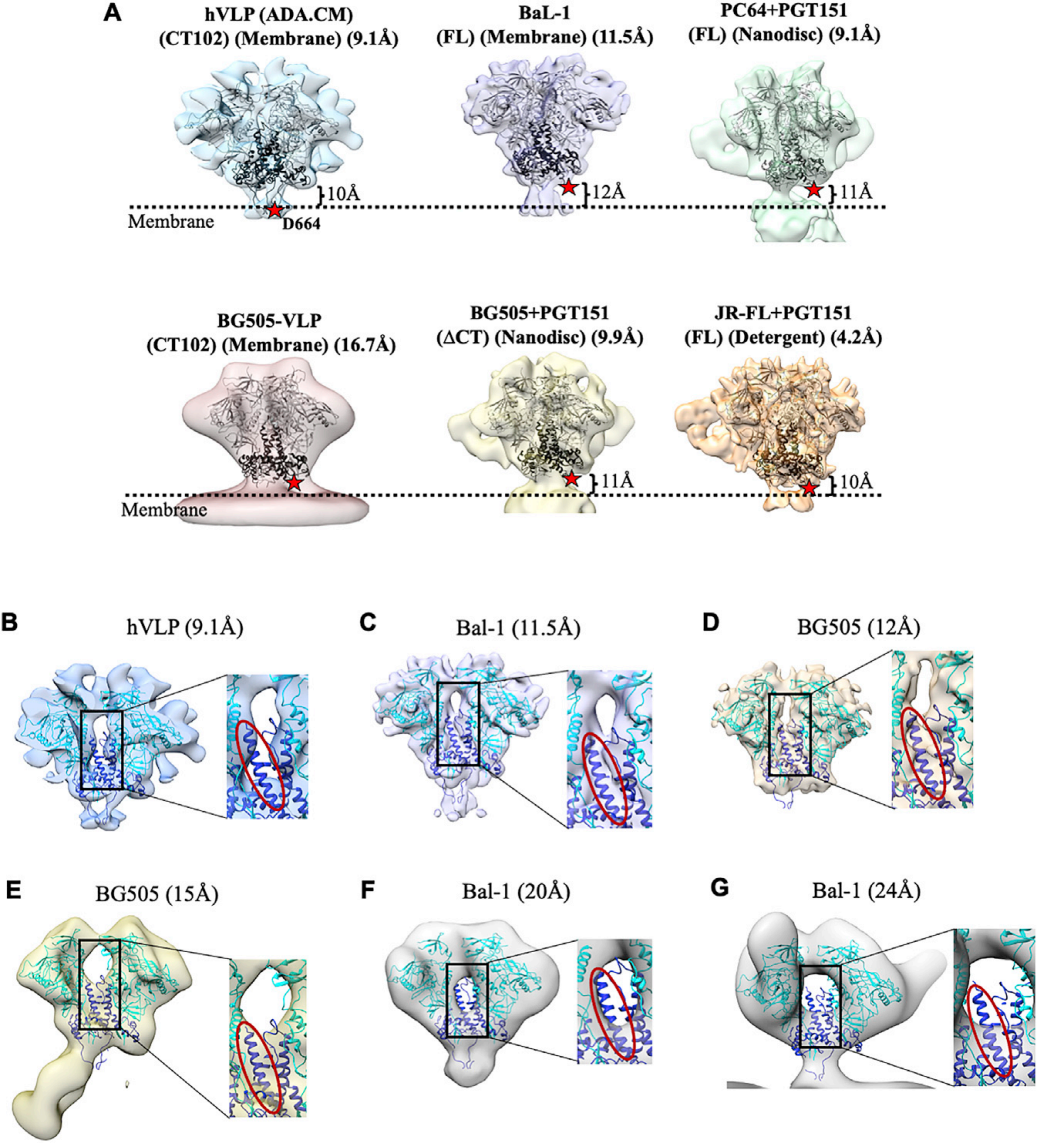
Comparison of Env structures with respect to position from membrane surface and HR1-C density, related to Figure 3 and 5 (A) Surface view of Env structure from different HIV strains: hVLP (ADA.CM), BaL-1, PC64, BG505 and JR-FL. Position of outer membrane surface as interpreted for each of the structures is indicated by a dotted black line. Position of Asp-664, which forms the end of HR2 helix region in gp41, is denoted by a red star in all structures. Low resolution structure of near full length BG505 in native membrane was obtained from VLPs displaying high levels of BG505 Env and having a CTD truncation similar to that in hVLP-Env. (B–G). Surface slab view of Env single particle cryo-EM and sub-tomogram averaged reconstructions with only 2 protomers shown to visualize HR1-C helix density clearly. In each panel, the central core region (black rectangle) is zoomed in and the HR1-C helix is indicated with red oval. Maps shown are (B) hVLP-Env, (C) BaL-1 (EMD-21412) (Li et al., 2020), (D) BG505 SOSIP.664 (EMD-5782) (Lyumkis et al., 2013), (E) BG505 SOSIP v5.2 (EMD-21075) (Cottrell et al., 2020), (F) BaL-1 (EMD-5019) (Liu et al., 2008), (G) BaL-1 (EMD-5457) (Tran et al., 2012).

The complete MPER peptide is unresolved in all reported structures of trimeric Env, owing to its membrane proximal location and/or possible variations in its conformation. Current knowledge of MPER structure is only derived from constructs without the Env ectodomain included (Fu et al., 2018; Piai et al., 2020). In a recently published structure of Env from BaL-1 virions, the MPER was indicated to form part of the stalk connecting Env ectodomain to the membrane (Li et al., 2020), which contrasts with the membrane-embedded MPER location identified in our structure (Figure S6). However, the embedded nature of the MPER in our map is in agreement with that inferred from other solubilized full-length Env structures and biophysical studies on binding of MPER-targeted antibodies to HIV-1 Env (Kim et al., 2011; Lee et al., 2016; Rantalainen et al., 2020; Wang et al., 2019). Analyzing Env structures from different HIV strains shows that the general position of the bulk of the ectodomain is ∼10–12 Å above the interpreted membrane surface in all cases (Figure S6). However, differences arise in the position of HR2 helix and subsequent MPER sequence leading to variability in MPER presentation and accessibility above the membrane among HIV-1 strains.

### A flexible gp41 stalk allows Env orientational freedom and MPER epitope exposure

**Figure 4:**
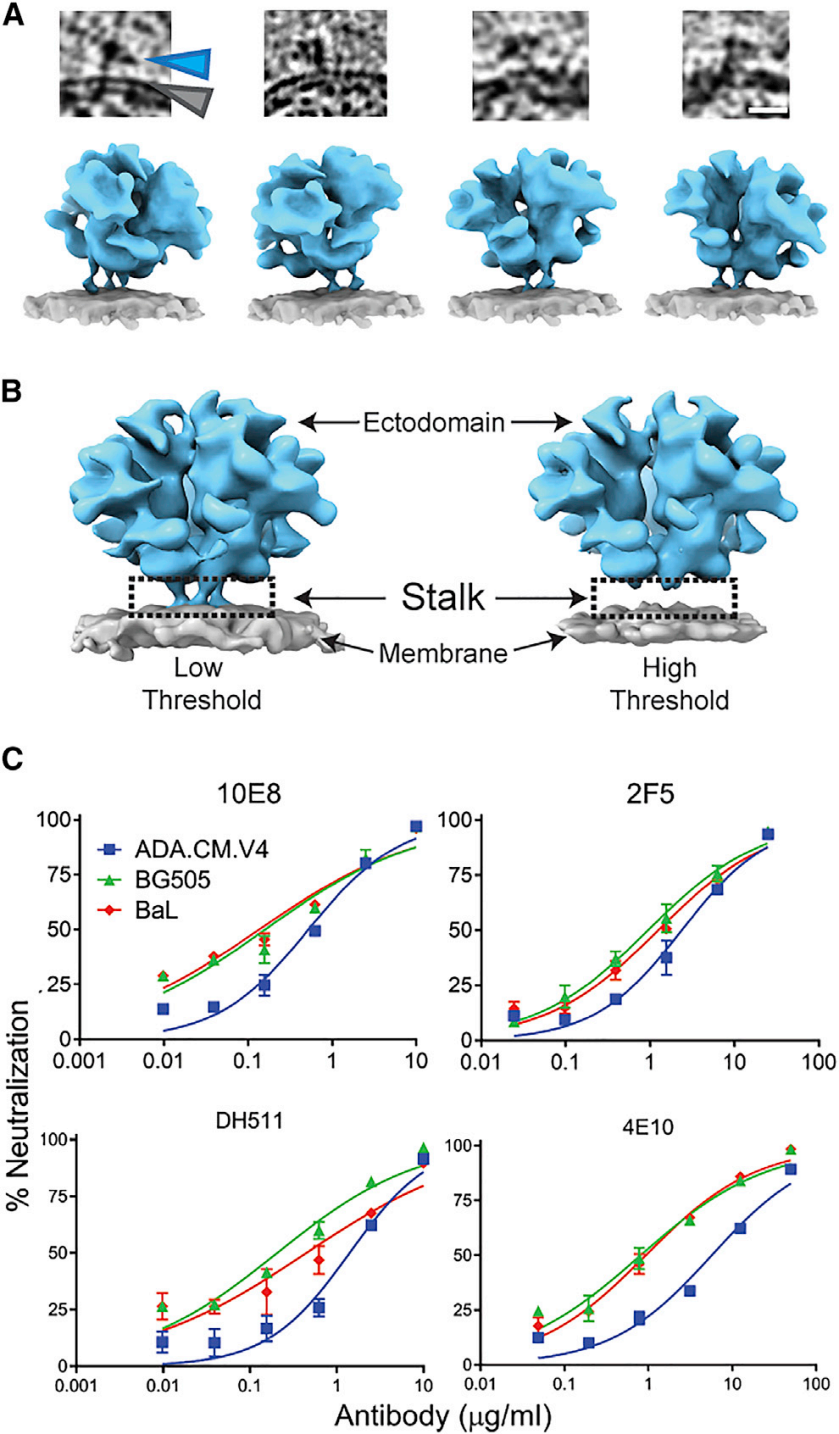
Flexible gp41 stalk in HIV Env leads to variation in MPER epitope accessibility (A) Top: tomogram slices showing examples of tilted Env on membrane surface. Scale bar equals 100 Å. Bottom: surface rendering of modeled Env structure (blue) in tilted orientations on membrane (gray) as seen in the tomograms. (B) Env density map rendered at low and high thresholds, respectively, showing loss of density in the stalk region (black rectangle). (C) Comparison of neutralization effect on different HIV-1 strains by MPER-directed bnAbs. Also see Figure S7 and Tables S2 and S3.

In our tomographic data, we observe tilting of hVLP-Env with respect to the membrane (Figure 4A), which leads to a lower density level for the stalk in our sub-tomogram-averaged map (Figure 4B). Thus, the tripod stalk of Env is flexible in its membrane-embedded state, allowing differential sampling of MPER and other membrane-proximal epitopes. Although tilting or lifting of Env from the membrane has been suggested based on bound complexes with MPER-targeted antibody 10E8 (Lee et al., 2016; Rantalainen et al., 2020) and gp120-gp41 interface-targeting antibody 35O22 (Huang et al., 2014), our results provide direct evidence of Env tilting on native membrane in an unliganded state.

**Figure S7:**
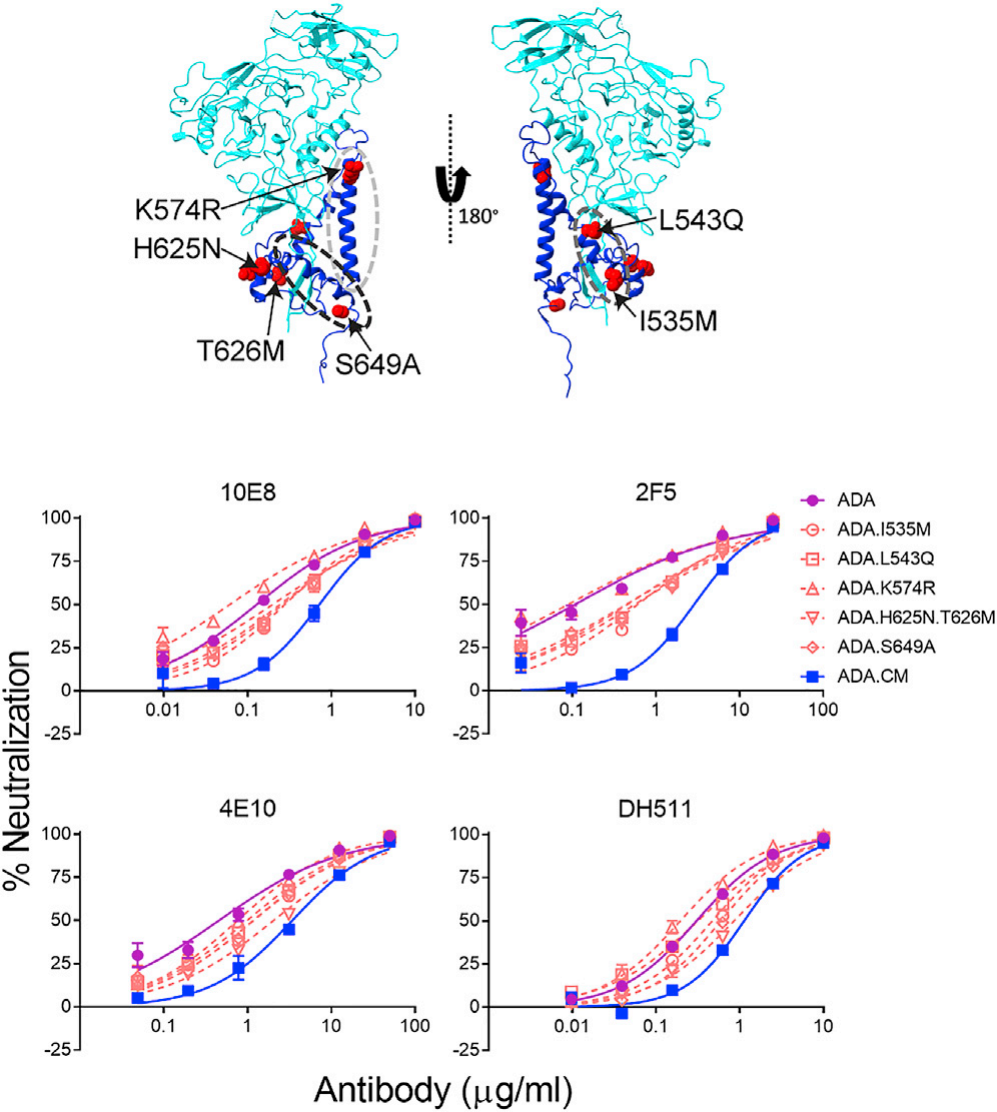
Neutralization effect of individual mutations in gp41 subunit of ADA.CM with respect to MPER-targeting antibodies, related to Figure 4 Top: Mutations relative to parental ADA virus in gp41 subunit that contribute to ADA.CM trimer hyperstability mapped onto its structure. The mutated residues are represented as red balls. HR1-C helix, HR2 helix and fusion loop proximal region are indicated by light gray, black and dark gray ovals respectively. Bottom: MPER antibody neutralization of single residue mutants of HIV-1 ADA in gp41 subunit, as indicated in the top panel. Each ADA point mutant corresponds to residues in ADA.CM that mutated during directed evolution of ADA to withstand destabilizing conditions (Leaman and Zwick, 2013). Curves shown are from a single experiment performed in duplicate.

Sensitivity to MPER bnAbs has been shown to be impacted by Env stability in its prefusion state (Kim et al., 2014), with increased neutralization observed after cellular receptor engagement (Chakrabarti et al., 2011; Frey et al., 2008). Based on the structural data discussed above, we envision that local structure of HR2, height of the Env ectodomain above membrane, and propensity of Env tilting have additional impact on Env sensitivity or resistance to MPER bnAbs. To test this hypothesis, we conducted comparative neutralization assays using MPER bnAbs against HIV-1 strains whose Env structures have been determined. As anticipated from the structural data, among the 3 viruses—ADA.CM hVLP, BaL-1, and BG505—ADA.CM was overall more resistant to MPER bnAbs (Figure 4C; Table S2). Neutralization and mutational analysis of proximal residues in ADA.CM that contribute to trimer stability (Figure S7) showed modest individual effects, but none were individually responsible for ADA.CM’s enhanced resistance to MPER bnAbs (Figure S7; Table S3).

### Structural variation in gp41’s HR1-C helical bundle suggests a conformationally variable central core

**Figure 5:**
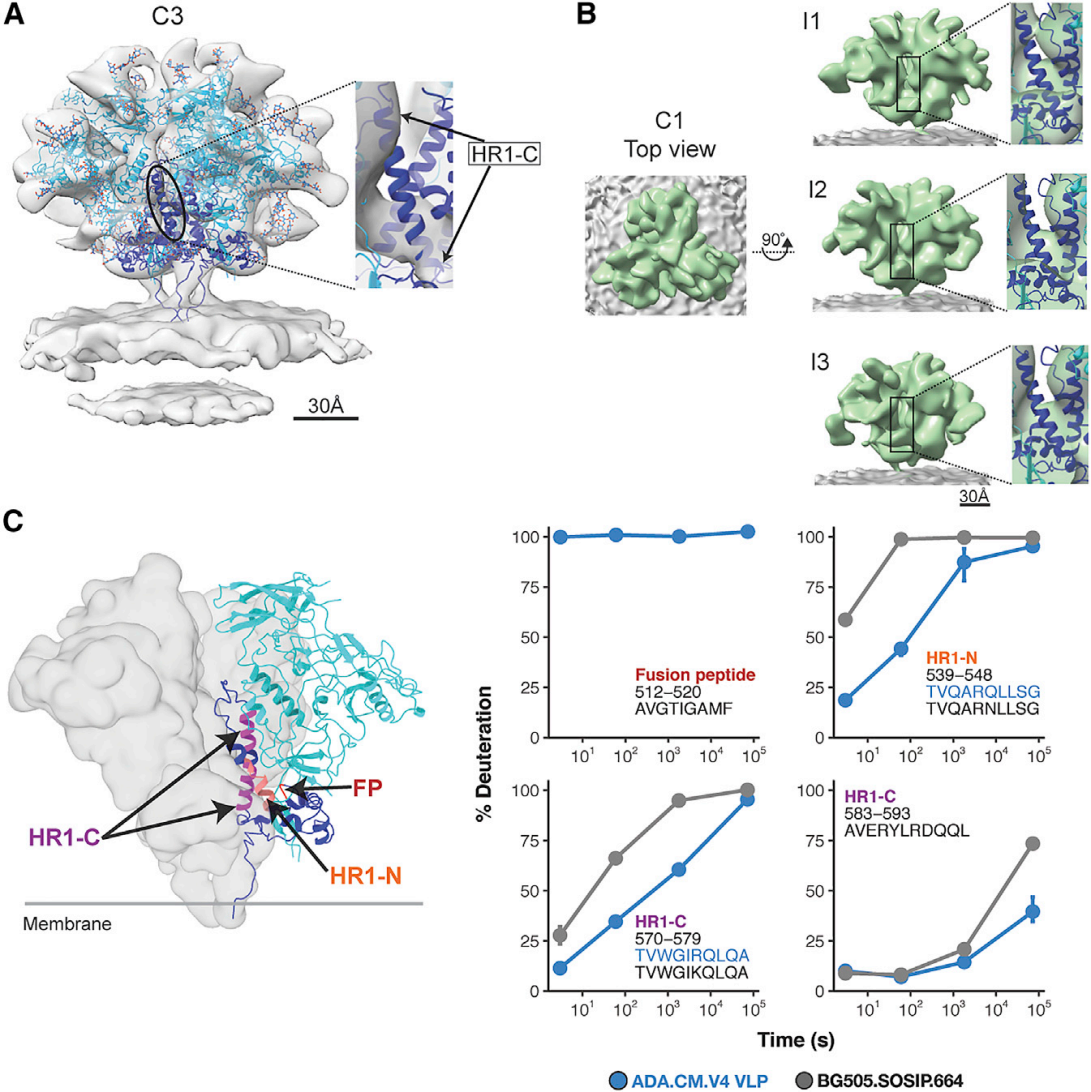
Conformational variation in central helical bundle (HR1-C) of hVLP-Env (A) Side-view of sub-tomogram-averaged Env map with fitted ribbon structure and colored similar to Figure 1. Zoomed inset shows fit of HR1-C helix into its respective density. See also Figure S6. (B) Top and side views of an asymmetric reconstruction of hVLP-Env with the three interfaces (I1, I2, and I3) of Env trimer shown along with magnified insets of fitted HR1-C helix. Black rectangle indicates the central core of the trimer where the HR1-C helix lies. See Figure S3 for resolution-related details. (C) HDX-MS deuterium uptake plots for fusion peptide (FP) and HR1 peptides (see also Figures S4 and S5). Surface rendering of Env was calculated using the fitted atomic model of hVLP-Env in (A) without glycans. Full FP region is not observed in hVLP-Env structure due to flexibility.

Another prominent difference in the gp41 subunit of hVLP-Env is absence of complete density in the averaged structure for the central C-terminal HR1 (HR1-Cs) helices (Figure 5A). The HR1-C helices comprised of amino acid residues 570–595 have been a hallmark of high resolution Env SOSIP structures with the central trimeric bundle composed of a HR1-C helix from each of the gp41 domains. However, in our structure, only partial density for the HR1-C helix can be observed corresponding to gp41 residues 570–577 and 582–595 (Figure 5A). Classification of Env sub-volumes with a tight cylindrical mask for the gp41 region did not yield any subset with higher density for HR1-C helices in our data. Considering that our sub-tomogram-averaged map has a local resolution range for the ectodomain between 5.7–12 Å (Figure S3), it is clear from comparisons with other cryoelectron microscopy (cryo-EM) maps at similar resolutions that the absence of complete HR1-C helix density in our structure is not an effect of map resolution (Figure S6).

To ascertain whether the observed HR1-C density was affected by applied symmetry to the map, we calculated an asymmetric structure of hVLP-Env at 10.7 Å resolution (Figure S3; Table 1). The asymmetric structure (Figure 5B) appears overall similar to the 3-fold symmetrized map (Figure 5A). Partial density for the HR1-C helices is consistently present across all 3 protomers in the asymmetric structure, with near-complete density for HR1-C observed in 1 protomer (Figure 5B).

HDX-MS analysis provides additional insight into the state of this key structural motif. Peptides in HR1-C helix (residues 570–579 and 583–593) are more protected in hVLPs than in BG505.SOSIP (Figure 5C), indicating increased backbone hydrogen bonding interactions in corresponding residues. Taken together, the sub-tomogram-averaged structures and HDX-MS analysis shows that the peptides in HR1-C region exist in a stable, ordered conformation in hVLP-Env even though they do not rigidly conform to Env’s global 3-fold structural symmetry.

### An extensive and heterogenous glycan shield in hVLP-Env protects critical epitopes

**Figure 6:**
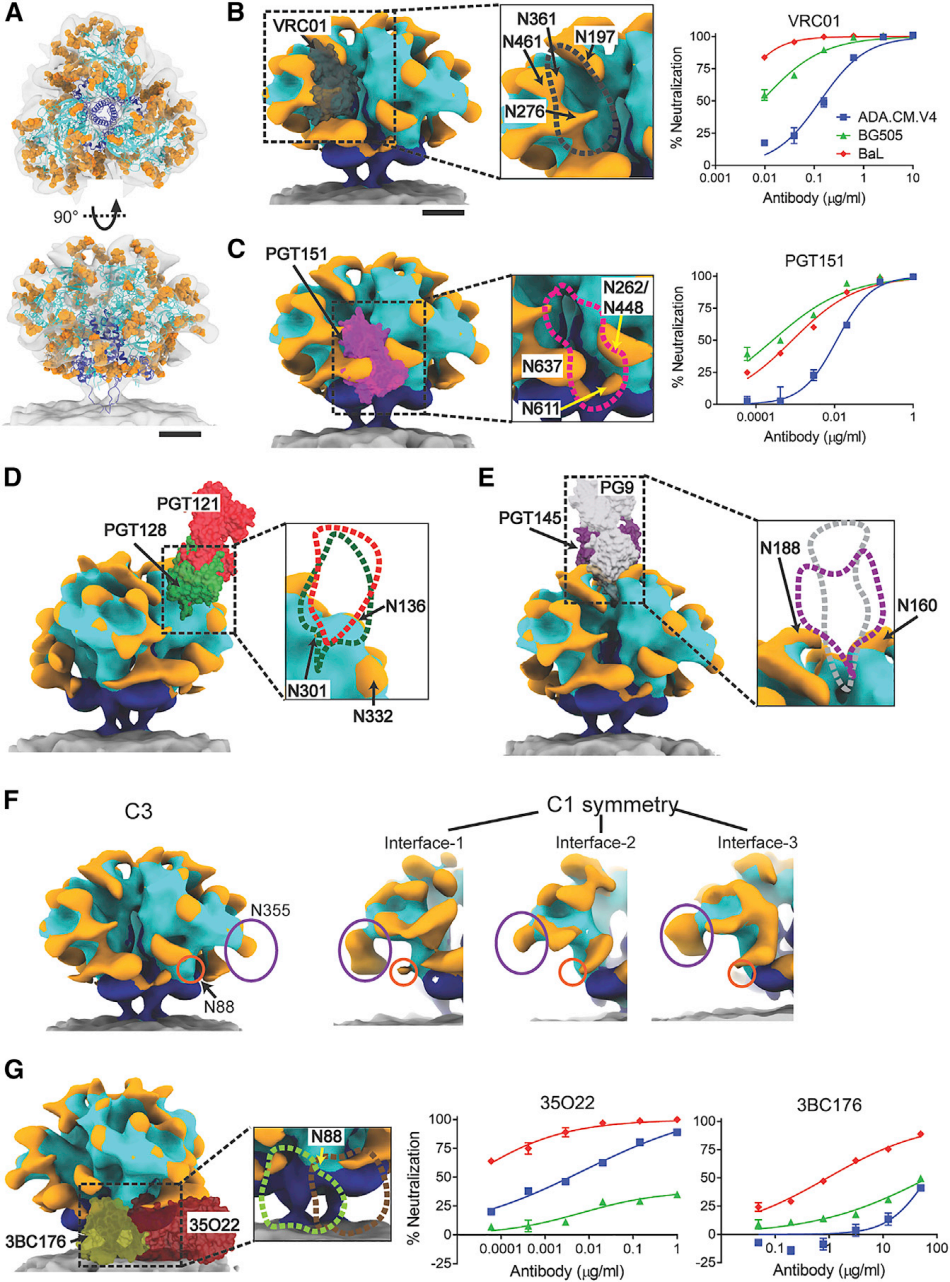
Presence of an extensive glycan shield in hVLP-Env (A) Top and side view of hVLP-Env with glycans rendered as orange ball-stick structures. Gp41 colored in dark blue and gp120 in cyan. (B–E and G) Binding positions of eight bnAbs—VRC01 (dark gray), PGT151 (pink), PGT128 (green), PGT121 (red), PG9 (light gray), PGT145 (purple), 35O22 (brown), and 3BC176 (green)—mapped onto hVLP-Env surface. gp120, gp41, glycan surfaces, and membrane are colored in cyan, dark blue, orange, and gray, respectively. Zoomed-in sub-panel outlines interaction surfaces corresponding to each bnAb. Glycans in the vicinity of bnAb binding sites are labeled. Neutralization of HIV-1 strains, ADA.CM.v4, BG505, and BaL in presence of bnAb are shown in (B), (C), and (G). (F) Differential glycosylation among trimer subunits in hVLP-Env represented using glycans at N88 (orange circle) and N355 (purple oval). Surface rendering of C3-symmetrized map on left followed by the 3 protomer interfaces from the C1 symmetry hVLP-Env map. Scale bars equal 30 Å. See also Tables S2 and S4.

The hVLP-Env structure displays distinguishable density features for N-linked glycans that decorate the ectodomain surface. In contrast to most available structures where density near to glycosylation sites often covers only the core n-acetylglucosamine (GlcNAc) sugars, in our unliganded hVLP-Env structure, extended density for glycan moieties is observed across the protein. Resolved density for multiple canonical glycan positions in gp120 and gp41 subunits extends beyond the range of current structural models, revealing the extent of the “glycan shield” (Figure 6A) (Wei et al., 2003). Glycans surrounding several key bnAb epitopes can be identified in the unliganded Env map, which reveal their orientations in the natural, antibody-naive state so as to occlude underlying epitopes.

At the CD4 receptor binding site (CD4bs) in hVLP-Env, overlaying the previously determined fragment antigen binding (Fab) structure of VRC01 (CD4bs bnAb) (Stewart-Jones et al., 2016) in its binding orientation highlights possible clashes with nearby glycans N197, N276, N361, and N461 (Figure 6B). These glycans have also been shown to overlap with the VRC01 epitope using molecular dynamic studies (Stewart-Jones et al., 2016). In hVLP-Env, extended density for these multiple glycans can be seen to clearly deter binding of CD4 epitope-directed nAbs (Figure 6B). These observations are corroborated in VRC01-based neutralization assays that show that hVLP-Env is substantially more resistant than BaL-1 Env (Figure 6B; Table S2), whose structure lacks extended glycosylation around this site (Li et al., 2020). Indeed, removal of N276 and N461 glycans has been demonstrated to facilitate engagement of germline precursor antibodies for VRC01-like nAbs that normally do not interact strongly with natively glycosylated Env (McGuire et al., 2013).

Similarly, long-range, extended densities for N-linked glycans are observed in the gp120 domain corresponding to residues N262 and N448, as well as in the gp41 domain corresponding to residues N611 and N637. The close spacing of N262/N448 to N611 likely orders these glycans to be visible in the averaged map (Figures 6A and 6C). Collectively, these glycans form a protective barrier over the fusion peptide (FP) in hVLP-Env (Figure 6C). Env’s FP is essential for viral cell entry but is surprisingly exposed to solution and highly dynamic by HDX-MS analysis (Figure 5C). Mapping the Fab structure of FP-targeting bnAb PGT151 (Lee et al., 2016) onto its corresponding epitope on hVLP-Env shows how the glycans would need to be displaced in order for the antibody to bind (Figure 6C). Though the ADA.CM hVLPs are neutralized by PGT151, they are relatively resistant compared to other strains (Figure 6C; Table S2), suggesting that glycan occlusion may impact neutralization by FP-targeted bnAbs as has been reported (Stewart-Jones et al., 2016).

Notably, we do not observe strong clashes with glycans at the V3-loop and V1/V2-loop antibody-binding super-sites. Overlaying the Fab structures of V3-loop targeting antibodies PGT121 (Garces et al., 2015) and PGT128 (Lee et al., 2015a) shows that the Fabs fit snugly between observed densities of glycans in that region, particularly N332, N136, and N301 (Figure 6D). Presence of an extended N301 glycan might provide some hindrance to binding of PGT128 Fab, but we only observe minimal ordered density for glycosylation at N301 in our map, which limits further analysis. Similarly, when the Fab structures of V1/V2-loop targeting antibodies PG9 (Wang et al., 2017) and PGT145 (Lee et al., 2017) were mapped onto the sub-tomogram-averaged Env map, no clashes were observed with nearby glycans at positions N160 and N188 (Figure 6E). Antibodies to the V3-loop and V1/V2-loops generally rely on extensive contacts with neighboring glycans for their activity. As can be seen from our analysis, absence of occlusion of their respective binding sites by the surrounding glycans might be an additional factor for these sites to be targeted efficiently by antibodies (Andrabi et al., 2015; Moyo et al., 2020).

Extensive N-linked glycosylation densities also persist in the asymmetric structure of hVLP-Env. Strikingly, we observe variations in glycan densities between different protomers, indicating heterogeneity in glycan occupancy. For example, the N355 glycan is present at all 3 protomers in the asymmetric Env reconstruction, but its density varies with respect to size and orientation at this site (Figure 6F). Similarly, the conserved N88 glycan, which forms part of the epitope of bnAb 35O22 (Huang et al., 2014) and borders that of 10E8 (Lee et al., 2016), can only be observed in 1 protomer, but not in others (Figure 6F), and is absent in the C3-symmetrized map. In MS analysis of hVLP-Env, the glycan site at N88, along with several others, appears to be occupied by a heterogenous population of complex sugars (Table S4). Although the observed heterogeneity in glycan processing of hVLP-Env (Table S4) is similar to previous reports (Berndsen et al., 2020; Cao et al., 2018; Guttman et al., 2014), our comparative structural analysis on asymmetric and symmetric hVLP-Env directly resolves glycan heterogeneity within protomers of the same Env trimer.

When comparing HIV bnAbs affected by both the presence of glycan and membrane proximity, binding of bnAb 35O22 (Huang et al., 2014) is highly dependent on the presence of N88 glycan, whereas 3BC176 binding (Lee et al., 2015b) is partially inhibited by it. Mapping binding orientation of both these Fabs onto hVLP-Env results in steric clashing of the Fab regions with the membrane surface (Figure 6G). Nevertheless, hVLPs are effectively neutralized by 35O22 but show relative resistance to 3BC176 (Figure 6G; Table S2), presumably due at least in part to 3BC176’s lower binding capacity (Lee et al., 2015b). Thus, a combination of the heterogenous presence of N88 glycan and tilting of hVLP-Env on the membrane is consistent with the differential access of 35O22 and 3BC176 to their epitopes on membrane-embedded Env.

### Conclusion

In this study, we have analyzed the structure of HIV particles displaying functional, membrane-bound Env. The ADA.CM Env trimers used are highly stable in a membrane environment, although they notably do not form well-ordered gp140 trimers in solution using the typically employed “SOSIP” mutations (Leaman and Zwick, 2013). This is true for the majority of Env sequences, which require extensive stabilizing modifications in order to encourage formation of stable ectodomain trimers (Rawi et al., 2020). Thus, the structural details discussed here may reflect features of Env strains that are not compatible with SOSIP modifications and thus have been resistant to structural characterization so far.

In hVLP-Env, we find substantial evidence that key parts of gp41, including the HR1 central helices and flexible stalk, are not rigidly fixed relative to the rest of the trimer. Indeed, root-mean-square deviation calculations among static high-resolution structures of full-length Env and SOSIP structures show higher deviation in the gp41 domain compared to gp120 domain (Table S5), despite the strains having greater than 75% sequence similarity (Table S6). Likewise, double electron-electron resonance (DEER) spectroscopy studies of SOSIP trimers have also shown a higher degree of variability in the gp41 domain (Stadtmueller et al., 2018), further substantiating the variable nature of gp41 as inferred from our structures.

Our data also demonstrate that even in membrane-associated Env, the FP is highly dynamic and exposed to solvent. Exposure of such a functionally critical and conserved component seems counterintuitive, but the dense clustering of glycans around the FP proximal region likely confers a degree of steric protection. However, breaches in the glycan shield, revealed by heterogeneity in glycan density in this region in our structures, can still offer access to the FP by bnAbs (Kong et al., 2016; Lee et al., 2016). Moreover, in cases where glycans form part of bnAb epitopes, heterogeneity in glycosylation provide mechanisms exploited by HIV-1 as a means of increasing epitope variability and facilitating evasion of antibody responses. Finally, the extent of ordered density between adjacent glycans in hVLP-Env suggests that the glycans are interacting in a stable manner, consistent with recent reports (Berndsen et al., 2020; Stewart-Jones et al., 2016).

By imaging Env in a membrane-bound context on VLPs, we found that, like other type-I fusion proteins such as SARS-CoV-2 S trimers (Ke et al., 2020), HIV-1 Env ectodomain sits atop a flexible stalk that affords it considerable freedom of motion to tilt relative to the membrane. This likely plays an important mechanistic role by affording fusion proteins the flexibility to bind receptors and refold during membrane fusion. The flexible stalk also impacts the accessibility of key epitopes that are sterically hindered on the membrane-facing side of the trimer ectodomain such as the FP, gp120/gp41 interface, and membrane-associated MPER. Thus, our structural analyses revealed previously uncharacterized Env features that impact presentation of prime epitopes that can help explain differences in neutralization sensitivity across diverse HIV-1 strains.

Lastly, our structural analyses of Env in immature viral particles show that Env is situated directly over 2-fold symmetric contacts of the Gag lattice, providing structural evidence of a direct physical Env-Gag interaction, helping to inform models for virion particle assembly and Env incorporation. Taken together, our results advance understanding of HIV-1 Env in the context of virion assembly, maturation, and conformational sampling, revealing insights that are generally unavailable through structural studies on recombinant proteins alone.

### Limitations of the study

The ADA.CM.v4 hVLPs, which are the primary samples used in our experiments, display Env that has a C-terminal truncation of 102 amino acids. This leaves about 40 amino acids of the CTD on the Env protein, sufficient to form part of the baseplate revealed in a recent nuclear magnetic resonance (NMR) structure (Piai et al., 2020). The loss of the rest of the tail could potentially have implications for Env stability, recruitment, and assembly in the VLPs as has been previously reported in studies of HIV (Chen et al., 2015) and related simian immunodeficiency virus (Affranchino and González, 2006; Celma et al., 2004; Fultz et al., 2001; Manrique et al., 2001). The hVLP sample was also treated with AT-2, a mild oxidizing agent, which inactivates HIV by causing the zinc finger domain of the nucleocapsid subunit to release zinc (McDonnell et al., 1997). However, it does not act directly on the Env protein, which our data and others’ have showed is intact in terms of structure, antigenicity, and cell receptor-mediated membrane fusion activity (Rossio et al., 1998; Rutebemberwa et al., 2007).

Our analyses of non-AT2-treated VLPs, produced in the presence of the HIV protease inhibitor darunavir and displaying truncated as well as full-length Env, showed that AT-2 treatment did not affect direct Env interactions with the underlying Gag lattice in the immature VLPs. In the mature hVLPs, the processed Gag core is not in direct contact with surface Env; however, we cannot rule out an indirect effect of AT-2 on nucleocapsid and the core. Lastly, a minor population of unprocessed Env (gp160 trimers) may exist, particularly on the VLPs bearing full-length Env, which cannot be distinguished from mature Env (gp120/gp41 heterotrimers) at the current resolution of our data. Despite the caveats above, in the range of immature particle examples we have examined, Env-MA-Gag interactions remain notably consistent, thus underscoring a persistent coupling between these key structural components of HIV.

## STAR★Methods

### Key resources table

**Table:** 

REAGENT or RESOURCE	SOURCE	IDENTIFIER
**Antibodies**		

Monoclonal anti-HIV-1 Env 4E10	Polymun	Cat# AB004; RRID:AB_2491029
Monoclonal anti-HIV-1 Env VRC01	Made in-house, Scripps Research	RRID:AB_2491019
Monoclonal anti-HIV-1 Env 10E8	Made in-house, Scripps Research	RRID:AB_2491067
Monoclonal anti-HIV-1 Env PGT151	Made in-house, Scripps Research (Falkowska et al., 2014)	N/A
Monoclonal anti-HIV-1 Env 2F5	Polymun	Cat# AB001; RRID:AB_2491015
Monoclonal anti-HIV-1 Env DH511	Made in-house, Scripps Research (Williams et al., 2017)	N/A
Monoclonal anti-HIV-1 Env 35O22	Made in-house, Scripps Research (Huang et al., 2014)	N/A
Monoclonal anti-HIV-1 Env 3BC176	Made in-house, Scripps Research	RRID:AB_2491063
Monoclonal anti-HIV-1 Env 8ANC195	William R Schief	RRID:AB_2491037
Monoclonal anti-HIV-1 Env PGDM1400	Made in-house, Scripps Research (Sok et al., 2014)	N/A
Monoclonal anti-HIV-1 Env PGT126	Made in-house, Scripps Research	RRID:AB_2491045
Monoclonal anti-HIV-1 Env 19b	Made in-house, Scripps Research (Moore et al., 1995)	N/A
Monoclonal anti-HIV-1 Env F105	Made in-house, Scripps Research (Posner et al., 1988)	N/A
Monoclonal anti-HIV-1 Env F240	Made in-house, Scripps Research (Cavacini et al., 1998)	N/A
Monoclonal anti-HIV-1 Env 98-6	Made in-house, Scripps Research (Taniguchi et al., 2000)	N/A
Monoclonal anti-Dengue virus DEN3	Dennis R Burton, Scripps Research (Hessell et al., 2009)	N/A
HRP- conjugated AffiniPure Goat Anti-Human IgG, Fc fragment specific	Jackson ImmunoResearch	Cat# 109-035-098; RRID: AB_2337586

**Chemicals, peptides, and recombinant proteins**		

Pro-Pro-Pro-Phe (PPPF)	Anaspec	N/A
Deuterium oxide 99.96%	Cambridge Isotope Laboratories	Cat# DLM-6-10X0.75
HybridSPE Phospholipid cartridge, 30mg bed wt.	Sigma-Aldrich	Cat# 55261-U
*n*-Dodecyl-beta-Maltoside Detergent (DDM)	ThermoFisher Scientific	Cat# 89902
25 kDa Polyethylenimine (PEI)	Polysciences	Cat# 23966
Darunavir protease inhibitor	https://aidsreagent.org/	ARP-11447
Dulbecco’s Modified Eagle Medium (DMEM)	ThermoFisher	Cat# 10313-021
Fetal Bovine Serum (FBS)	ThermoFisher	Cat# 10437-028
L-Glutamine	ThermoFisher	Cat# 25030-081
Penicillin Streptomycin	ThermoFisher	Cat# 15140-122
Puromycin	ThermoFisher	Cat# A11138-03
Trypsin-EDTA	ThermoFisher	Cat# 25200-056
Freestyle 293 Expression Medium	ThermoFisher	Cat# 12338-018
OptiPrep Density Gradient Medium (iodixanol)	Sigma	Cat# D1556
DEAE-dextran hydrochloride	Sigma	Cat# D9885
Bright-Glo luciferase reagent	Promega	Cat# E2620
Aldrithiol-2 (AT-2)	Sigma	Cat# 143049
One-step Ultra TMB Substrate	ThermoFisher Scientific	Cat# 34028
T-20 (N-acetylated derivative; Enfuvirtide acetate salt)	https://aidsreagent.org/	Cat# ARP-12732

**Deposited data**		

Sub-tomogram averaged EM density map: HIV Env with C3 symmetry	This study	EMD-25178
Sub-tomogram averaged EM density map: HIV Env with C1 symmetry	This study	EMD-25186
Sub-tomogram averaged EM density map: HIV Env from immature virus-like particles with C1 symmetry	This study	EMD-25809
Fitted coordinates of HIV-1 Env	This study	PDB ID: 7SKA
Density map of BaL-1 Env in complex with VRC01 IgG	(Tran et al., 2012)	EMD–5457
Density map of BaL-1 Env	(Liu et al., 2008)	EMD-5019
Density map of BG505 SOSIP v5.2	(Cottrell et al., 2020)	EMD-21075
Density map of BG505 SOSIP.664	(Lyumkis et al., 2013)	EMD-5782
Sub-tomogram averaged density map of Env BaL-1	(Li et al., 2020)	EMD-21412
Density map of immature Gag-CA hexamer	(Mattei et al., 2018)	EMD–0164
Atomic coordinates of HIV-Env	(Pan et al., 2020)	PDB ID: 6ULC
Atomic coordinates of HIV-Env TMD-CTD	(Piai et al., 2020)	PDB ID: 6UJV
Atomic coordinates of immature Gag-CA hexamer	(Schur et al., 2015)	PDB ID: 4USN
Atomic coordinates of Gag-MA	(Hill et al., 1996)	PDB ID: 1HIW

**Experimental models: Cell lines**		

ADA.CM.v4 (aka ADA.CM.755^∗^.v4) in HEK239T stable cell line	Made in-house, Scripps Research (Stano et al., 2017)	N/A
BG505.755^∗^ in HEK293T stable cell line	This study	N/A
TZM-bl (CD4^+^.CCR5^+^.CXCR4^+^) cells	https://aidsreagent.org/	ARP-8129
HEK293T	ATCC	Cat# CRL-3216, RRID: CVCL_0063
Expi293F	Thermo-Fisher Scientific	Cat# A14527
Freestyle293F	Thermo-Fisher Scientific	Cat# R79007

**Recombinant DNA**		

pSG3ΔEnv Env-deficient HIV-1 backbone plasmid	https://aidsreagent.org/	Cat# ARP-11051
pLentiIII.ADA.CM	Made in-house, Scripps Research (Stano et al., 2017)	N/A
pcDNA.ADA	Made in-house, Scripps Research (Leaman and Zwick, 2013)	N/A
pcDNA.ADA.CM	Made in-house, Scripps Research (Leaman and Zwick, 2013)	N/A
pcDNA.ADA.I535M	Made in-house, Scripps Research (Leaman and Zwick, 2013)	N/A
pcDNA.ADA.L543Q	Made in-house, Scripps Research (Leaman and Zwick, 2013)	N/A
pcDNA.ADA.K574R	Made in-house, Scripps Research (Leaman and Zwick, 2013)	N/A
pcDNA.ADA.H625N.T626M	Made in-house, Scripps Research (Leaman and Zwick, 2013)	N/A
pcDNA.ADA.S649A	Made in-house, Scripps Research (Leaman and Zwick, 2013)	N/A
pSVIII-BG505	https://aidsreagent.org/	Cat# ARP-11518
pSVIII-BaL-1	https://aidsreagent.org/	Cat# ARP-11445
pPI4-BG505.SOSIP.664	(Sanders et al., 2013)	N/A

**Software and algorithms**		

Leginon	(Carragher et al., 2000)	N/A
SerialEM	(Mastronarde, 2005)	https://bio3d.colorado.edu/SerialEM/
Motioncor2	UCSF	https://emcore.ucsf.edu/ucsf-software
IMOD 4.10.15	(Kremer et al., 1996)	https://bio3d.colorado.edu/imod/
Relion 2.1	(Bharat and Scheres, 2016; Scheres, 2012)	https://www3.mrc-lmb.cam.ac.uk/relion/index.php/Main_Page
PEET 1.12	(Nicastro et al., 2006)	https://bio3d.colorado.edu/PEET/
EMAN2	(Chen et al., 2019)	https://blake.bcm.edu/emanwiki/EMAN2/e2tomo
COOT	MRC	https://www2.mrc-lmb.cam.ac.uk/personal/pemsley/coot
UCSF Chimera	UCSF	https://www.cgl.ucsf.edu/chimera/
UCSF ChimeraX	UCSF	https://www.rbvi.ucsf.edu/chimerax/
ImageJ	(Schneider et al., 2012)	https://imagej.nih.gov/ij/
HDExaminer v2	Sierra Analytics	http://massspec.com/hdexaminer/
DriftScope	Waters	https://www.waters.com/nextgen/us/en.html
Byonic	Protein Metrics	https://proteinmetrics.com/byos/
HX-Express v2	(Guttman et al., 2013)	N/A

### Resource availability

#### Lead contact

Further information and requests for resources and reagents should be directed to and will be fulfilled by the lead contact, Dr. Kelly K. Lee (kklee@uw.edu).

#### Materials availability

All unique/stable reagents generated in this study are available from the lead contact without restriction. The hVLPs will be provided upon reasonable request by a materials transfer agreement (MTA).

### Experimental models and subject details

#### Cell lines for producing VLPs

The stable HEK293T cell line expressing high levels of HIV Env, ADA.CM.v4, was described previously (Stano et al., 2017). Cells were grown in DMEM supplemented with 10% heat-inactivated fetal bovine serum (FBS; ThermoFisher Scientific), 20 mM L-glutamine, 100 U/mL penicillin, 100 μg/mL streptomycin, and 2.5 μg/mL puromycin (all media additives from ThermoFisher Scientific) and incubated at 37þC with 5% CO_2_. A stable cell line expressing Env BG505 was generated using a similar method, namely by transduction of HEK293T cells using a lentiviral vector (pLentiIII.BG505.755^∗^) containing the Env gene BG505 with a stop codon at position 756. Transduced cells were subjected to two rounds of sorting by FACS using fluorescently labeled quaternary neutralizing antibody PGT145 and non-neutralizing CD4BS antibody b6, then gating cells for PGT145^high^ b6^low^. The gender of the HEK293T cell line is female.

#### Cell lines for neutralization assays

HEK293T cells (ATCC) were transfected to produce pseudovirus used for neutralization assays and virus ELISAs. TZM-bl cells (NIH ARRRP) were used as the target cells for HIV infection in neutralization assays. Both cell lines were cultured in DMEM (ThermoFisher Scientific) supplemented with 10% fetal bovine serum, 20 mM L-glutamine, 100 U/mL penicillin, and 100 μg/mL streptomycin (all additives from ThermoFisher Scientific) and incubated at 37þC with 5% CO_2_.

#### Cell lines for antibody production

Freestyle 293F cells (ThermoFisher Scientific) used for expression of antibodies were grown at 37þC, 8% CO_2_, with shaking at 125 rpm in Freestyle 293 media (ThermoFisher Scientific) without additives. The cell lines were not authenticated within our lab, however a certificate of analysis from the manufacturer is available. The gender of the 293F cell line is female.

#### Cell lines for BG505.SOSIP production

Expi293F cells (ThermoFisher Scientific) used for expression of BG505.SOSIP Env glycoproteins were grown at 37þC, 8% CO_2_, with shaking at 125 rpm in Freestyle 293 media (ThermoFisher Scientific) without additives. The cell lines were not authenticated within our lab, however a certificate of analysis from the manufacturer is available. The gender of the Expi293F cell line is female.

### Method details

#### hVLP expression and purification

To generate hVLPs, stable cell lines expressing high levels of ADA.CM.v4 Env were transfected using Env-deficient HIV-1 backbone plasmid pSG3ΔEnv (NIH ARRRP) and 25 kDa PEI (Polysciences) as a transfection reagent. Immature hVLPs and full-length ADA pseudovirus were produced by adding the protease inhibitor darunavir (NIH AIDS Reagent Program) to transfected cells at the same time as the DNA and PEI to a final concentration of 2 μM. Supernatants were harvested 3 days after transfection and cleared by centrifugation at 3,000 × *g* for 15 min. hVLPs were pelleted at 80,000 × *g* for 1 h and resuspended 100-fold concentrated in PBS. VLPs were separated from cellular debris using iodixanol density gradient centrifugation. Here, concentrated hVLPs were overlaid on a 9.6 to 20.4% iodixanol (Optiprep; Sigma) gradient, formed by layering iodixanol in 1.2% increments, and centrifuged at 200,000 × *g* for 1.5 h at 4°C in an SW41Ti rotor (Beckman). Fractions (1 mL each) were collected starting from the top and fractions 6-9 were pooled. Purified hVLPs were brought up to 15 mL with PBS and concentrated to ∼0.2 mL using a 100 kDa MWCO Amicon centrifugal filter (Millipore). VLPs were inactivated by adding aldrithiol-2 (AT-2) to a final concentration of 2.5 mM and samples were incubated at RT for 2 h. The volume was again increased to 15 mL with PBS and hVLPs were concentrated using a 100 kDa MWCO centrifugal filter to 250-fold the concentration in the original transfection supernatant. BG505-VLPs were produced using stable cell lines expressing Env BG505, similar to the hVLPs as described above. Env copy number on BG505 VLPs was notably lower than on ADA.CM.v4 hVLPs. Additionally, BG505 VLPs were also more fragile, which precluded extensive cryo-ET data collection and HDX-MS analysis.

#### Antibody expression and purification

Freestyle 293F cells at a density of 1 × 10^6^/mL were co-transfected using 4.0 kDa PEI MAX (Polysciences) with equal amounts of heavy and light chain plasmids and pAdVAntage protein expression enhancing vector (Promega), using 0.5 μg of plasmid per 1 mL of culture. Five days post-transfection the supernatant was harvested, and antibodies were purified using Protein A Sepharose 4B (ThermoFisher Scientific) and eluted using 0.2 M Citric Acid, pH 2.5. Protein was buffer exchanged into PBS and stored at 4þC.

#### BG505.SOSIP expression and purification

Soluble BG505.SOSIP protein was expressed and purified as previously described (Verkerke et al., 2016). Briefly, Expi293F cells were grown to a density of 3 × 10^6^/mL prior to co-transfection with pPI4-BG505.SOSIP.664 and furin protease expression plasmid in a 3:1 ratio using PEI MAX (Polysciences). Six to seven days post-transfection the supernatant was cleared by filtration and the SOSIP purified by *Galanthus nivalis* lectin affinity, followed by ion-exchange and hydrophobic interaction chromatography. Trimeric product was buffer exchanged into PBS and stored at 4°C.

#### Cryo-ET sample preparation and tilt-series acquisition

Purified AT-2 treated hVLP samples were mixed with 10nm gold beads (Aurion BSA Gold Tracer 10nm) at a ratio of 15:1 (v/v). Using a Vitrobot Mark IV (FEI Co.), 3 μL of this mixture was applied to glow discharged, C-Flat 1.2/1.3, 200mesh or 400mesh grids (Electron Microscopy Sciences), blotted for 4-5 s and plunge frozen in liquid ethane. For the non-AT-2 treated immature ADA.CM VLPs and immature full-length ADA VLPs, purified sample was mixed with 10nm gold beads at a ratio of 10:1; 3 ul of this mixture was applied to glow-discharged 400 mesh Cu Quantifoil R 1.2/1.3 grids (Electron microscopy sciences) and plunge frozen in liquid ethane. Vitrobot was maintained at 4°C and 100% humidity during all these experiments.

For the AT-2 treated hVLPs, frozen grids were imaged using a 300 kV Titan Krios with a Gatan K2 direct electron detector and GIF energy filter with slit width of 20 eV. Tilt-series were collected in a dose-symmetric tilting scheme from −54° to +54° or from −48° to +48° with a step size of 3° using Leginon (Carragher et al., 2000) or SerialEM softwares (Mastronarde, 2005). Tilt-series were collected in counting mode at a magnification of 53000X, corresponding to a pixel size of 2.58 Å per pixel. The total dose per tilt series was ∼64-68 e^-^/Å^2^. A total of 526 tilt-series were collected across multiple sessions.

For the non-AT-2 treated immature ADA.CM.v4 hVLPs and immature full-length ADA.CM pseudovirus, frozen grids were imaged on a 300 kV Titan Krios equipped with a Gatan K3 direct electron detector and a GIF energy filter with a slit width of 20 eV. Tilt series were collected from −60° to +60° with a step size of 3° using SerialEM (Mastronarde, 2005). A dose-symmetric tilt scheme was applied between −48° and +48°, and the remaining angles were collected with a bidirectional approach. Images were collected in super-resolution mode at a nominal magnification of 64,000X, corresponding to a pixel size of 0.6932 Å/pixel. The total cumulative dose was 94 e-/Å2. For the non-AT-2 treated immature full-length ADA.CM VLPs, a total of 39 usable tilt-series were collected. For the non-AT-2 treated immature ADA.CM.v4 hVLPs, 52 usable tilt-series were collected.

For the non-AT-2 treated immature full-length ADA.CM pseudovirus, additional data was also collected on a 200kV Glacios microscope with a K2-Summit direct electron detector. A total of 17 tilt-series were collected with a nominal magnification of 28000X, corresponding to a pixel size of 1.4975 Å/pixel. Tilt series were collected from −60° to +60° with a step size of 3° using SerialEM with up to 45° in dose-symmetric tilt scheme (Mastronarde, 2005). A total dose of 110 e-/Å^2^ was applied to each tilt-series.

#### Tomogram reconstruction

For the AT-2 treated hVLP dataset, tilt-series image frames were corrected for beam-induced motion using motioncor2 (Zheng et al., 2017). Using batch tomography via the Etomo interface in IMOD software package (Kremer et al., 1996; Mastronarde and Held, 2017), tilt-series were processed for tomogram generation using standard procedures. Tilt series images were aligned using gold bead markers and aligned tilt-series were used to generate a three-dimensional volume using weighted back-projection method. The final tomograms were rotated, binned, and low pass–filtered for visualization. Tilt-series with non-optimal alignments were discarded. In the end, 423 tilt-series were selected for further processing.

For immature ADA.CM VLPs and immature full-length ADA VLPs, Images were motion-corrected using motioncor2 (Zheng et al., 2017) and tomograms were reconstructed and CTF corrected in EMAN2 using default parameters (Chen et al., 2019).

#### Sub-tomogram averaging of hVLP-Env

##### Initial model generation

Eight tilt-series were randomly selected. CTF estimation and correction for these were done using the Ctfplotter program (Xiong et al., 2009). Tomograms were then generated for these CTF-corrected tilt-series in IMOD at the unbinned pixel size (Kremer et al., 1996; Mastronarde and Held, 2017). The aligned tilt-series, reconstructed tomograms, file listing the tilt angles and electron dose values were imported into Relion software suite (Scheres, 2012) according to its conventions (Bharat and Scheres, 2016). Using the 3dmod graphical user interface (Kremer et al., 1996), 2067 Env particles on the surface of hVLPs were manually picked. The picked particle coordinates were imported into Relion (Scheres, 2012) and corresponding sub-volumes extracted. These were then subjected to 3D classification with a spherical mask covering small parts of the membrane using C1 and C3 symmetry with no initial model provided. Both the C1 and C3 symmetry classifications resolved a 3D class that looked similar to the expected structure of HIV-1 Env. The 3D class from the C3 symmetry run showing clear Env density contained a total of 811 sub-volumes and was selected as initial model.

#### C3-symmetrized hVLP-Env structure

Tilt-series were imported into EMAN2’s sub-tomogram averaging pipeline (Chen et al., 2019). 1k X 1k tomograms were generated within EMAN2 using default parameters. The binned tomograms were then used for semi-automated particle picking in PEET software (Nicastro et al., 2006). Consolidated particles from the semi-automated picking were further curated manually using the 3dmod interface (Kremer et al., 1996) to remove wrongly positioned particle points such as those that were in the membrane or on the inside of VLPs. Curated particle coordinates (63592 particles) were then imported into EMAN2 through the e2spt_boxer.py interface (Chen et al., 2019). CTF-estimation for all the tilt-series and subsequent CTF-corrections were carried out within EMAN2. Sub-volumes were initially extracted at 4xbinning corresponding to a pixel size of 10.32 Å per pixel. Sub-tomogram refinement was carried using a spherical mask including Env on surface and a part of the membrane. The 3D structure previously generated in Relion (Bharat and Scheres, 2016; Scheres, 2012) was used as initial model after low pass filtering to 60 Å. Sub-volumes were then re-extracted at 2X binning using the particle orientations from the 4X binned refinement output. Sub-tomogram refinement was repeated using the 2X binned data with a cylindrical mask covering only the ectodomain and outer membrane layer. Particle orientations were locally refined within a 30° angular limit from the positions calculated in the 4Xbin refinement. Sub-volumes closer than 120 Å when measured between centers were removed. Sub-volumes with low cross-correlation scores were also removed according to EMAN2’s default settings. Remaining particles were then re-extracted at the original, un-binned pixel size and re-refined starting from previously determined orientations at 2X binning. Sub-tilt refinement in EMAN2 was then carried out using default parameters following sub-tomogram refinement with un-binned sub-volumes (Chen et al., 2019). For sub-tilt refinement, a threshold mask was used that enclosed only the Env ectodomain portion without any membrane density. The threshold mask was generated using the mask creation tool in the Relion package (Scheres, 2012). The final masked ectodomain map contained 32802 sub-volumes with a calculated resolution of 9.13 Å at 0.143 FSC cut-off value.

#### Asymmetric hVLP-Env map

The initial sub-volumes used for generation of the asymmetric map were the same as that used for the C3 symmetrized map described above. Refinement strategies were also nearly identical between the two structures except the asymmetric map was generated with C1 symmetry. However, the refinement and processing steps were carried out completely independent of each other. The number of sub-volumes in the final C1 hVLP-Env structure was 29074 with a global resolution of 10.67 Å at 0.143 FSC cut-off.

#### Structure of hVLP-Env and Gag layer from immature virions

A total of 1520 sub-volumes from only immature virions were used to generate C3 and C1 symmetrized maps of membrane bound Env using 2Xbinned data in EMAN2 (Chen et al., 2019) by similar procedures as described above.

In the unmasked maps, a third density layer was observed underneath the membrane bilayer. Relaxing symmetry of the C3-density map to C1, showed a more defined organization in the Gag layer. Hence, a short, cylindrical mask enclosing only the Gag layer was used for local refinement of the Gag layer using C1 symmetry, starting from the final refined positions derived from the C3-symmetrized immature Env map. This focused refinement gave rise to a 23 Å map of the Gag protein layer (0.143 FSC cut-off). The refined Gag-CA density map was fitted back into the Gag-CA layer of the relaxed to C1 symmetry full Env map for further analyses.

#### Sub-tomogram averaged structure of BG505-Env from VLPs

Purified BG505-VLPs were mixed with 10nm gold beads (Aurion BSA Gold Tracer 10nm) at a ratio of 15:1 (v/v). The mixture was applied to C-Flat grids and plunge frozen using a Vitrobot Mark IV (FEI Co.) similar to the procedure used for hVLPs above.

Frozen grids were imaged using a 300 kV Titan Krios with a Gatan K2 direct electron detector and GIF energy filter with slit width of 20 eV. Tilt-series were collected in a dose-symmetric tilting scheme from −54° to +54° with a step size of 3° using Leginon (Carragher et al., 2000) software. Tilt-series were collected in counting mode at a magnification of 53000X, corresponding to a pixel size of 2.58 Å per pixel. The total dose per tilt series was ∼64-68 e^-^/Å^2^. A total of 49 tilt-series were collected.

Tilt-series were imported into EMAN2’s sub-tomogram averaging pipeline (Chen et al., 2019). 1k X 1k tomograms were generated within EMAN2 using default parameters. Particle points were picked manually in the e2spt_boxer.py interface (Chen et al., 2019). Further refinement and processing steps were carried out similar to the hVLP-Env sub-tomogram averaging procedures described above. Briefly, sub-volumes were initially extracted at 4xbinning corresponding to a pixel size of 10.32 Å per pixel. Sub-tomogram refinement was carried using a spherical mask including Env on surface and a part of the membrane. The initial model generated for hVLP-Env in Relion (Bharat and Scheres, 2016; Scheres, 2012) was used as initial model for BG505-Env also with low pass filtering to 60 Å. Sub-volumes were then re-extracted at 2X binning using the particle orientations from the 4X binned refinement output. Sub-tomogram refinement was repeated using the 2X binned data with a cylindrical mask covering only the ectodomain and outer membrane layer. Particle orientations were locally refined, and duplicates were removed. Sub-tilt refinement in EMAN2 was carried out using default parameters (Chen et al., 2019). The final map contained 2773 sub-volumes with a calculated resolution of 16.7 Å at 0.143 FSC cut-off value.

#### Model fitting and glycan modeling

Full length Env structure from strain 92UG037.8 purified with detergents (PDB ID: 6ULC) (Pan et al., 2020) was fitted as a rigid body into the 9.13 Å resolution hVLP-Env map. Each protomer, comprising of gp120-gp41 heterodimer, was fitted individually into the map using UCSF Chimera (Pettersen et al., 2004). The unstructured loop region at the end of gp41, comprising of amino acid residues 654-664, was fitted into the clearly delineated stalk density using ‘Flexible fitting’ feature in Coot software (Emsley et al., 2010). For modeling glycan moieties, previously published high-resolution Env structures, PDB IDs: 5FUU and 5FYJ-L, containing well-built glycan models were used as reference (Lee et al., 2016; Stewart-Jones et al., 2016). Appropriate glycan chains from these structures were copied into corresponding glycan positions in the hVLP-Env map based on the presence of unoccupied density. In regions where the unoccupied density was larger than the modeled glycan chains available from the reference PDB structures, these extra densities were left un-modeled.

The final model of hVLP-Env, as generated above, was used for rigid body fitting into the other sub-tomogram averaged maps of asymmetric hVLP-Env and immature hVLP-Env. All rigid body fitting procedures were carried out using UCSF Chimera (Pettersen et al., 2004).

Root mean square deviation (RMSD) calculations between pairs of HIV Env structures was carried out using UCSF Chimera (Pettersen et al., 2004). Multiple sequence alignments were carried out using the Clustal Omega web service (Madeira et al., 2019).

Structure and tomographic images were generated using UCSF Chimera (Pettersen et al., 2004), ChimeraX (Pettersen et al., 2021), IMOD’s graphical user interface (Kremer et al., 1996) and ImageJ (Schneider et al., 2012).

#### Neutralization assays

ADA.CM.v4 hVLPs were prepared for neutralization assays as described above except omitting the AT-2 inactivation step. Pseudotyped viruses were similarly produced by co-transfection of HEK293T cells using pSG3ΔEnv backbone plasmid and Env-complementation plasmid. Serial dilutions of antibodies were added to virus and the mixture was incubated for 1 h at 37°C prior to addition to TZM-bl target cells. DEAE-dextran was added to wells to a final concentration of 10 μg/mL. After incubating for 72 h at 37°C, cells were lysed, Bright-Glo luciferase reagent (Promega) was added, and luminescence was measured using a Synergy H1 microplate reader (Bio-Tek).

#### Virus ELISA

ELISAs on directly immobilized hVLPs were performed as previously described (Tong et al., 2012). Briefly ADA.CM.v4 hVLPs (prepared as detailed above and treated with or without AT-2) were immobilized on microwell plates at a 20x concentration for 2 h at 37þC. Plates were blocked with 4% non-fat dry milk (NFDM) PBS for 1 h at 37þC, probed with serial dilutions of primary antibodies and, subsequently, goat anti-human-Fcɣ-HRP secondary antibody (Jackson) in PBS + 0.4% NFDM, and with washes using PBS between each step (detergent was omitted from all steps). Signal was developed using One-step Ultra TMB Substrate (ThermoFisher).

#### Light microscope imaging of infected cells

TZM-bl target cells were plated in 96-well plates (100 μl at 1 × 10^5^/mL), and 24 h later an equal volume of hVLPs were overlaid on the cells. After a 72 h incubation, cells were imaged using a Evos FL light microscope (Life Technologies) and 20x objective.

#### Hydrogen-deuterium mass-spectroscopy (HDX-MS) of ADA.CM.v4 particles and BG505.SOSIPs

##### HDX-MS using quench lysis method

Frozen stocks of ADA.CM.v4 VLP containing approximately 0.5mg/mL envelope protein or purified BG505.SOSIP diluted to the same concentration were buffer-exchanged into HEPES-buffered saline (HBS, 10mM HEPES-KOH, pH 7.4, 150mM NaCl) using Zeba Spin columns with a 7kDa MWCO (ThermoFisher Scientific). 10μL of VLP solution was diluted with 90μL of deuterated HBS (HEPES Buffer Saline, pH 7.4) for the indicated labeling time. The reaction was quenched and lysed by addition of an equal volume of 0.2% formic acid, 4M guanidine hydrochloride, 0.2M TCEP (tris(2-carboxyethyl)phosphine), 0.2% DDM (n-Dodecyl-B-D-maltoside), followed by 30 s incubation on ice. 20μL of a 300mg/mL solution of ZrO_2_ beads (HybridSPE-Phospholipid) (Adhikary et al., 2017) in 0.1% formic acid was then added to the solution, followed by vortexing for 30seconds on ice. The bead/protein mixture was then moved to a 0.22μm cellulose acetate centrifuge filter tube (Spin-X, Corning) and spun for 30 s at 13,000xG, 0°C. The flow-through was transferred to a thin-wall PCR tube and frozen in liquid nitrogen. Samples were thawed on ice and passed over a custom packed pepsin column (2.1 × 50 mm) kept at 15°C with a flow of 0.1% trifluoroacetic acid (TFA), 2% acetonitrile (ACN) at 200 μL/min for 5 min.

Digested peptides were collected on a Waters XSelect CSH C18 XP VanGuard Cartridge, 130Å, 2.5 μm, 2.1 mm X 5 mm before separation on a Waters ACQUITY UPLC Peptide CSH C18 Column, 130Å, 1.7 μm, 1 mm X 100 mm using a gradient of 7 to 14% B over 1 min, 14 to 30% B over 12.5 min, and 30 to 50% B over 1 min, followed by washing with three rapid gradients between 95 and 5% B (A: 0.1% formic acid, 0.025% trifluoroacetic acid, 2% acetonitrile; B: 0.1% formic acid in 100% acetonitrile). The liquid chromatography system was coupled to a Waters Synapt G2-Si Q-TOF with ion mobility enabled. Source and de-solvation temperatures were 70°C and 130°C respectively. The StepWave ion guide settings were tuned to prevent non-uniform gas phase proton exchange in the source (Guttman et al., 2016).

During the separation step, a series of 250 μL injections were used to clean the pepsin column: (1) Fos-choline-12 (Anaspec) in 0.1% TFA; (2) 2 M GuHCl in 0.1% TFA; (3) 10% acetic acid, 10% ACN and 5% IPA (Hamuro and Coales, 2018; Majumdar et al., 2012). After each gradient, the trap column was washed with a series of 250 μL injections: (1) 10% FA; (2) 30% trifluoroethanol; (3) 80% MeOH; (4) 66% 2-propanol, 34% ACN; (5) 80% ACN (Fang et al., 2011).

#### HDX-MS using solution digestion method

Env on hVLPs was quantified by SDS-PAGE using BG505 SOSIPs as a standard. HDX-MS reactions were initiated by diluting 20 uL of either hVLPs or BG505 SOSIPs with 180 uL deuteration buffer (10 mM Phosphate, 150 mM NaCl, 85% D_2_O (Cambridge Isotope Laboratories)) to a final pH = 7.45. Samples were deuterated for 5 s, 60 s, 30 min, or 3 h before being diluted 1:1 with ice cold quench buffer (8 M urea, 200 mM TCEP [tris(2-carboxyethyl) phosphine] and 0.2% formic acid (FA)) to a final pH of 2.5. Quenched samples were digested with 30 ug/mL of porcine pepsin (Worthington Labs) under quench conditions for 5 min on ice. Labeled peptides were purified by high-speed centrifugation at 0°C (2 min at 25,000 rcf) and immediately flash frozen in liquid nitrogen. BG505 SOSIP samples were handled identically to ensure consistent labeling and back exchange. Frozen samples were stored at −80°C until analysis.

Samples were thawed for 5 min on ice and manually injected into a custom built HDX LC system kept at 0°C using a 500 uL sample loop. Samples were trapped on a Waters ACQUITY UPLC CSH C18 VanGuard 130Å, 1.7 μm, 2.1 mm by 5 mm trap column for 7 min with a flow of solvent A [2% acetonitrile, 0.1% FA, 0.025% trifluoroacetic acid (TFA)] at a rate of 150 μL/min. Peptides were resolved over a Waters ACQUITY UPLC CSH C18 130Å, 1.7 μm, 1 × 100 mm column using a 20 min linear gradient of 3% to 50% solvent B (Solvent B: 100% acetonitrile and 0.1% FA) and analyzed using Waters Synapt G2-Si Q-TOF as described above. Following each injection, the sample loop and trap were washed as described above.

Deuterium uptake analysis was performed with HD-Examiner (Sierra Analytics) and HX-Express v2. For solution digestion experiments data was extracted using CDCReader and analyzed using HXExpress v2 (Guttman et al., 2013) with binomial fitting and bimodal deconvolution.

Internal exchange standards Pro-Pro-Pro-Ile [PPPI] and Pro-Pro-Pro-Phe [PPPF] were included in each reaction to control for variations in ambient temperature during the labeling reactions (Zhang et al., 2012). Back-exchange was measured by including bradykinin peptide at 2μg/mL in the deuterated buffer to serve as a fully deuterated control. The back-exchange level ranged from 12%–16% across experiments; deuterium uptake was not corrected.

Totally deuterated (TD) samples were prepared by collecting purified peptide eluent following reverse phase LC separation of a pepsin digested undeuterated sample. Following evaporation of the LC elution buffer the peptides were resuspended in HDX PBS pH 7.50 Buffer, deuterated in deuteration Buffer for 1 h at 65°C and quenched and frozen as described above.

For peptide identification, un-deuterated peptides were collected from the LC system, dried by speed-vac, and resuspended in 5% acetonitrile, 0.1% formic acid for re-injection on an Orbitrap Fusion for MS/MS using EThcD fragmentation. Data was analyzed using Byonic (Protein Metrics) and manually compared to undeuterated sample data.

#### Peptic digest analysis

Peptic digest products were collected from the HDX chromatography system, dried by speed-vacuum, and resuspended in aqueous buffer for nanoscale liquid chromatography (nanoLC-MS) using a 90-min linear gradient from 2%–40% acetonitrile. Products were analyzed on an Orbitrap Fusion mass spectrometer (ThermoFisher Scientific) using a high-energy collisional dissociation (HCD) product-dependent electron-transfer/high-energy collision dissociation (EThcD) method with a targeted mass list method with a targeted mass list using HexNAc, HexHexNAc, and Hex2HexNAc m/z (204, 366, and 528 respectively) to trigger EThcD.

Glycopeptide data were visualized and processed by Byonic (Version 3.8, Protein Metrics Inc.) using a 10 ppm precursor and 10 ppm fragment mass tolerance. Glycopeptides were searched using the N-glycan 309 mammalian database in Protein Metrics PMI-Suite and scored based on the assignment of correct c- and z- fragment ions. The true-positive entities were further validated by the presence of glycan oxonium ions m/z at 204 (HexNAc ions) and 366 (HexNAcHex ions).

### Quantification and statistical analysis

Neutralization assays shown in Figures 6, S2, and S7 were repeated two or more times for each of two separate virus preparations. Data shown are from a representative experiment. Results were plotted and non-linear regression performed using GraphPad Prism software. Error bars show the standard deviation. Virus ELISAs were performed individually and were repeated to verify results. Data shown are from a representative experiment and analysis was performed the same way as for neutralization assays.

Hydrogen/deuterium-exchange mass spectrometry experiments were performed in triplicate (quench lysis method) or duplicate (solution digestion method) from separate particle preparations. Deuterium uptake levels shown in Figures 3, 5, and S5 were determined using HD-Examiner (Sierra Analytics) or HX-Express v2 (Guttman et al., 2013), with the error bars reflecting the standard deviation of the technical replicates. Mass spectral analysis to test for the presence of bimodal spectra was performed with HX-Express v2.

## Data Availability

• Sub-tomogram averaged density maps with corresponding atomic models have been deposited to the Electron Microscopy DataBase and the Protein Data Bank respectively. These are publicly available as of the date of publication. Accession numbers are listed in the key resources table. • This paper does not report original code. • Any additional information required to reanalyze the data reported in this paper is available from the lead contact upon request.
