# Supplementary material for: Cryo-ET of Env on intact HIV virions reveals structural variation and positioning on the Gag lattice
Source: Cell. 2022 Feb 17;185(4):641–653.e17. doi: 10.1016/j.cell.2022.01.013 (PMC9000915; doi:10.1016/j.cell.2022.01.013)
Supplement: Document S1. Supplemental tables S1–S6 [file mmc1.pdf]

Cell, Volume *185*

Supplemental information

Cryo-ET of Env on intact HIV virions

reveals structural variation

and positioning on the Gag lattice

Vidya Mangala Prasad, Daniel P. Leaman, Klaus N. Lovendahl, Jacob T. Croft, Mark A. Benhaim, Edgar A. Hodge, Michael B. Zwick, and Kelly K. Lee

Supplemental Tables:

Table S1.

Half-maximal inhibitory concentration (IC_{50}) values for antibody neutralization of VLPs displaying Envs of full-length ADA.CM, ADA.CM with truncation matching that of hVLPs (ADA.CM.755*) and high Env displaying hVLPs (ADA.CM.V4). Related to Figure 1 and S1.

		ADA.CM	ADA.CM.755*	ADA.CM.V4 (hVLP)
IC_{50} (μg/ml)	VRC01	0.11	0.11	0.19
	T-20	0.020	0.020	0.024
	10E8	0.37	0.26	0.52
	DH511	0.71	0.89	1.12
	2F5	2.06	2.31	3.39
	4E10	1.87	3.57	4.64
	PGT151	0.0042	0.0032	0.0099
	35O22	0.0093	0.0019	0.0032
	8ANC195	2.51	2.10	2.98

Table S2.

IC_{50} values for neutralization of HIV-1 strains by MPER-targeted antibodies and Gp120-Gp41 interface/glycan targeting antibodies. Related to Figures 4 and 6.

		ADA.CM.V4	BG505	BaL
	MPER-targeting antibodies			
IC_{50} (µg/ml)	10E8	0.46	0.16	0.21
	DH511	1.26	0.21	1.00
	2F5	2.88	0.94	1.36
	4E10	5.16	0.81	1.83
	Gp120-gp41 interface and glycan targeting antibodies			
	VRC01	0.16	0.0086	0.0028
	PGT151	0.011	0.00026	0.00083
	35O22	0.0032	>20	0.00018
	3BC176	66.7	36.5	0.93

Table S3.

IC_{50} values for neutralization of single point mutants in gp41 of ADA by MPER-targeted antibodies. Related Figure 4 and S7.

	IC_{50} (μg/ml)					
	VRC01	T20	10E8	DH511	2F5	4E10
ADA	0.31	0.016	0.13	0.31	0.11	0.46
ADA.I535M	0.36	0.013	0.28	0.47	0.60	1.17
ADA.L543Q	0.39	0.072	0.24	0.32	0.46	0.99
ADA.K574R	0.27	0.024	0.061	0.20	0.089	0.76
ADA.H625N.T626M	0.41	0.11	0.26	0.86	0.56	2.05
ADA.S649A	0.29	0.021	0.20	0.60	0.44	1.18
ADA.CM	0.16	0.013	0.72	1.13	2.86	3.43

Table S4.

N-linked glycosylation detected in hVLP-Env through peptide digest analysis by mass spectrometry. Related to Figure 6.

Asn (N) residue numbers	Complex sugars[1]	Oligo-mannose[1]	Hybrid sugars[1]
88	HexNAc(4)Hex(5)<br>HexNAc(5)Hex(5)<br>HexNAc(5)Hex(4)<br>HexNAc(4)Hex(5)Fuc(1)	HexNAc(2)Hex(5)<br>HexNAc(2)Hex(4)	HexNAc(3)Hex(6)<br>HexNAc(3)Hex(5)Fuc(1)
136	HexNAc(5)Hex(6)NeuAc(3)<br>HexNAc(6)Hex(6)Fuc(2)NeuAc(2)<br>HexNAc(6)Hex(7)Fuc(1)NeuAc(2)		
187/188	HexNAc(5)Hex(3)Fuc(2)		
197	HexNAc(3)Hex(4)Fuc(2)NeuAc(1)		
262		HexNAc(2)Hex(9)<br>HexNAc(2)Hex(8)	
276	HexNAc(3)Hex(6)<br>HexNAc(3)Hex(5)<br>HexNAc(3)Hex(6)NeuAc(1)<br>HexNAc(3)Hex(5)NeuAc(1)	HexNAc(2)Hex(7)<br>HexNAc(2)Hex(6)<br>HexNAc(2)Hex(5)	
295-301		HexNAc(2)Hex(9)<br>HexNAc(2)Hex(5)	

332-334*	HexNAc(6)Hex(6)NeuAc(1)<br>HexNAc(5)Hex(5)NeuAc(2)<br>HexNAc(6)Hex(6)NeuAc(3)<br>HexNAc(6)Hex(6)NeuAc(2)		
339	HexNAc(6)Hex(6)Fuc(2)NeuAc(2)<br>HexNAc(5)Hex(6)Fuc(3)NeuAc(2)		
355	HexNAc(6)Hex(4)		
361	HexNAc(5)Hex(5)NeuAc(2)<br>HexNAc(6)Hex(6)NeuAc(2)		
448	HexNAc(5)Hex(5)Fuc(2)NeuAc(1)<br>HexNAc(6)Hex(6)Fuc(2)NeuAc(2)<br>HexNAc(6)Hex(7)Fuc(3)NeuAc(4)	HexNAc(2)Hex(9)	
461/462	HexNAc(5)Hex(5)<br>HexNAc(5)Hex(5)Fuc(1)<br>HexNAc(5)Hex(4)Fuc(1)<br>HexNAc(4)Hex(5)Fuc(1)NeuAc(1)<br>HexNAc(5)Hex(6)Fuc(1)NeuAc(1)<br>HexNAc(4)Hex(5)Fuc(1)		
616	HexNAc(6)Hex(6)Fuc(2)NeuAc(1)		
637			HexNAc(3)Hex(6)

* un-glycosylated peptide also observed

[1] Abbreviations and glycan classifications according to *Essentials of Glycobiology*, *3rd ed.*

HexNAc: N-acetylhexosamines; Hex: Hexose; Fuc: L-Fucose; NeuAc: N-acetyl neuraminic acid

Table S5.

Backbone carbon chain RMSD[1] in angstroms for gp120 and gp41 subunits in high resolution Env glycoprotein structures. Related to Figure 3 and 5.

**Gp120**	92UG037.8 full length (PDB: 6ULC)	JR-FL full-length (PDB: 5FUU)	AMC011 full length (PDB: 6OLP)	BG505 SOSIP (PDB: 4ZMJ)
92UG037.8		2.204	2.980	1.752
JR-FL			1.654	1.758
AMC011				2.924
BG505				
**Gp41**	92UG037.8	JR-FL	AMC011	BG505
92UG037.8		5.105	4.888	3.908
JR-FL			1.928	2.063
AMC011				1.299
BG505				

[1]root mean square deviation

Table S6.

Percentage sequence similarity in Env amongst HIV-1 strains compared in Table S6. Related to Figure 3 and 5.

	ADA-CM	Bal-1	JR-FL	92UG037.8	BG505
ADA-CM	100.0	84.39	88.82	76.19	76.97
Bal-1		100.0	89.78	74.68	77.29
JR-FL			100.0	76.52	76.04
92UG037.8				100.0	81.07
BG505					100.0
